# Biochemical and anatomical leaf characteristics of oak trees contribute to differences in photosynthetic capacity between leaf habits

**DOI:** 10.1093/aobpla/plaf063

**Published:** 2025-11-03

**Authors:** Mina Momayyezi, Kyra A Prats, Andrew J McElrone, Morgan E Furze

**Affiliations:** Department of Viticulture and Enology, University of California, Davis, One Shields Avenue, Davis, CA 95616, United States; Department of Botany and Plant Pathology, Purdue University, 915 Mitch Daniels Blvd, West Lafayette, IN 47907, United States; Center for Plant Biology, Purdue University, 915 Mitch Daniels Blvd, West Lafayette, IN 47907, United States; Department of Viticulture and Enology, University of California, Davis, One Shields Avenue, Davis, CA 95616, United States; USDA-ARS, Crops Pathology and Genetics Research Unit, 425 Storer Mall, Davis, CA 95616, United States; Department of Botany and Plant Pathology, Purdue University, 915 Mitch Daniels Blvd, West Lafayette, IN 47907, United States; Center for Plant Biology, Purdue University, 915 Mitch Daniels Blvd, West Lafayette, IN 47907, United States; Department of Forestry and Natural Resources, Purdue University, 715 Mitch Daniels Blvd, West Lafayette, IN 47907, United States

**Keywords:** leaf anatomy, leaf habit, micro-CT, oaks, photosynthetic capacity, phylogenetic comparative methods

## Abstract

Since the leaf is the primary site of photosynthesis, leaf habit impacts the period over which a plant can acquire carbon. However, leaf biochemistry and anatomical characteristics that contribute to differences in photosynthetic capacity between leaf habits deserve further attention. Using a comparative framework, we examined photosynthetic capacity between oak species (*Quercus* spp.) with different leaf habits. We performed gas exchange measurements and micro-computed tomography imaging of leaves to compare their biochemical and anatomical characteristics between evergreen and deciduous oak species and to link these leaf characteristics as drivers of photosynthetic capacity. Deciduous species had higher photosynthetic capacity than evergreen. Deciduous leaves had higher maximum carboxylation rate of Rubisco, maximum rate of electron transport, and rate of triose phosphate utilization than evergreen leaves. Their higher photosynthetic capacity was also influenced by leaf anatomical characteristics. Deciduous leaves had more densely packed mesophyll, a greater portion of palisade than spongy mesophyll, and a higher mesophyll surface area than evergreen leaves. Overall, our work suggests that greater investment in leaf structures such as densely packed palisade mesophyll facilitates higher photosynthetic capacity in deciduous species and helps compensate for their shorter growing season.

## Teaser

We used a comparative framework to demonstrate how the biochemistry and internal anatomy of deciduous oak leaves support greater photosynthetic capacity than evergreen oak leaves. Deciduous oaks invest more in leaf structures for light absorption that facilitate higher photosynthetic capacity to compensate for their shorter growing season than evergreen oaks.

## Introduction

Evergreen and deciduous represent two extremes of the leaf habit spectrum with leaves exhibiting opposite responses to seasonal changes in environmental conditions (e.g. winter temperatures, summer drought) and inherently different resource allocation strategies (Chabot and Hicks 1982, Givnish 2002, Van Ommen Kloeke et al. 2012). Deciduous leaves cope with these environmental stressors through seasonal shedding and avoidance, while evergreen leaves persist year-round. Even though the persistence of leaves provides evergreen species with a longer period during which they can be photosynthetically active, they must then also contend with the respiratory maintenance of keeping the same leaves alive throughout unfavourable times of the year (Kikuzawa 1991) as well as endure additional stresses like herbivory (Peñuelas and Llusià 2004). Furthermore during cold periods, evergreen species may also undergo photoinhibition and cuticular abrasion, consequently reducing their physiological activity (Koppel and Heinsoo 1994, Lamontagne et al. 1998, Corcuera et al. 2005). The structural investment of evergreen leaves may favour intracellular airspaces to accommodate ice and protect against frost damage (Wyka and Oleksyn 2014). In contrast, the investment in defence is much less for short-lived deciduous leaves, and their direction of structural investment may be more towards building a leaf that optimizes photosynthetic activity and performance (Mooney and Dunn 1970; Wright et al. 2004 ). Deciduous leaves generally compensate for a shorter growing season by having higher photosynthetic rates per unit mass than evergreen ones (Givnish 2002).

The oaks (genus *Quercus*) have evolved evergreen and deciduous leaf habits multiple times (Cavender-Bares 2019), highlighting their capacity to inhabit a range of environments and providing a unique system to comparatively study the leaf biochemistry and anatomy of related oak species with different leaf habits. Previous work in this genus has linked leaf habit with leaf carbon physiology whereby deciduous oak species were evolving towards higher non-structural carbohydrates—the primary products of photosynthesis—than their evergreen relatives (Furze et al. 2021). Higher non-structural carbohydrate storage of deciduous oaks could result from a higher photosynthetic capacity compared to evergreen oaks. Indeed, other studies have shown that during favourable conditions, deciduous species had higher photosynthetic rates than evergreen (Reich et al. 1998, Givnish 2002, Wright et al. 2004, Edwards et al. 2017), including studies specifically investigating oaks (Takashima et al. 2004, Brüggemann et al. 2009, Baldocchi et al. 2010, Koller et al. 2013, Peguero-Pina et al. 2016, Alonso-Forn et al. 2021). However, there remains an opportunity to explore photosynthetic capacity across the genus *Quercus* using phylogenetic comparative methods to disentangle the influence of relatedness and leaf habit. Further, studying how biochemical and anatomical characteristics of leaves contribute to potential differences in photosynthetic capacity between leaf habits will advance our understanding of evolutionary trade-offs (e.g. leaf life span versus photosynthetic capacity) and help address broader questions about whether structural and biochemical investments in leaves reflect evolutionary pressures and functional constraints.

The photosynthetic capacity of leaves can be influenced by biochemical capacity, CO_2_ diffusion through stomata and mesophyll, and anatomy of the leaf itself (Grassi and Magnani 2005). The biochemical and diffusive limitations that govern photosynthetic rates can be measured through photosynthetic CO_2_ response curves [*A*_n_−*C*_i_; net assimilation (*A*_n_) vs. intercellular airspace CO_2_ concentration (*C*_i_)] (Long and Bernacchi 2003, Sharkey 2016). An *A*_n_−*C*_i_ curve derives the maximum carboxylation rate of the enzyme Rubisco (*V*_cmax_), the maximum rate of electron transport (*J*_max_), and the rate of triose phosphate utilization (TPU), thus determining the biochemical limitations of photosynthetic reactions within the leaf (see Table 1 for definitions of parameters and their abbreviations). The biochemical limitations of photosynthesis are also inherently linked to the anatomy of leaves, as ease of CO_2_ diffusion inside the leaf [i.e. mesophyll conductance (*g*_m_)] is determined/driven in part by leaf structural characteristics such as the surface area of the whole mesophyll [*SA*_mes(S+P)_] exposed to the intercellular airspace (IAS) volume, the IAS volume as a fraction of the total mesophyll volume also known as mesophyll porosity (θ_IAS_), and the ratio of spongy mesophyll volume to palisade mesophyll volume (S:P) (Flexas et al. 2008, Flexas et al. 2012, Théroux-Rancourt and Gilbert 2017, Evans 2021, Théroux-Rancourt et al. 2021). Any inherent variation in leaf structural and physiological characteristics, as a function of seasonality for the leaf habit, may play an important role in regulating photosynthetic capacity in oak species. For example, previous work showed that deciduous leaves of *Quercus kelloggii* had higher mesophyll porosity than evergreen leaves of *Quercus suber* (Borsuk et al. 2022), which may influence photosynthetic capacity. While the aforementioned studies suggest that deciduous oaks have higher photosynthetic rates than evergreen (Reich et al. 1998, Givnish 2002, Wright et al. 2004, Edwards et al. 2017), recent work showed that Mediterranean sclerophyllous evergreen oaks had rates similar to congeneric deciduous species due to leaf anatomical traits (i.e. increased mesophyll surface area) that offset limitations imposed by their higher leaf mass per area (LMA) (Peguero-Pina et al. 2017).

**Table 1.** Summary of abbreviations and definitions for biochemical, diffusional, anatomical, and morphological parameters estimated on leaves

Abbreviation	Definition
*A*_n_ (µmol m^−2^ s^−1^)	Net assimilation rate
*A*_c_ (µmol m^−2^ s^−1^)	Assimilation rate at the Rubisco limiting state
*A*_j_ (µmol m^−2^ s^−1^)	Assimilation rate at the RuBP regeneration limiting state
*A*_p_ (µmol m^−2^ s^−1^)	Assimilation rate at the triose phosphate utilization limiting state
*A*_max_ (µmol m^−2^ s^−1^)	Maximum assimilation rate
*C*_i_ (µmol mol^−1^)	Intercellular airspace CO_2_ concentration
*C*_a_ (µmol mol^−1^)	Ambient CO_2_ concentration inside LI-COR 6800 chamber
*C*_c_ (µmol mol^−1^)	Chloroplast CO_2_ concentration
ϕ_PSII_	Quantum yield of photosystem II
*J*_flu_ (µmol m^−2^ s^−1^)	Electron transport rate by chlorophyll fluorescence
*V*_cmax_ (µmol m^−2^ s^−1^)	Maximum carboxylation rate of the enzyme Rubisco
*J*_max_ (µmol m^−2^ s^−1^)	Maximum rate of electron transport (over *A*_n_−*C*_i_ curve at given irradiance)
TPU	Rate of triose phosphate
*R*_d_ (µmol m^−2^ s^−1^)	Respiration in the light
*g_s_* (mol m^−2^ s^−1^)	Stomatal conductance to CO_2_
*g*_m_ (mol m^−2^ s^−1^)	Mesophyll conductance
*SA*_mes(S+P)_ (µm^2^ µm^−3^)	Surface area of the whole mesophyll, determined by the ratio of the spongy and palisade mesophyll surface area to the whole mesophyll volume, equivalent to *S*_m_ as used in Théroux-Rancourt et al. (2023)
*SA*_mes(P)_ (µm^2^ µm^−3^)	Surface area of the palisade mesophyll, determined by the ratio of the palisade mesophyll surface area to the whole mesophyll volume
*SA*_mes(S)_ (µm^2^ µm^−3^)	Surface area of the spongy mesophyll, determined by the ratio of the spongy mesophyll surface area to the whole mesophyll volume
θ_IAS_ (m^3^ m^−3^)	Mesophyll porosity, determined by intercellular airspace (IAS) volume as a fraction of the total mesophyll volume
P:M	Palisade mesophyll volume to total mesophyll volume
S:M	Spongy mesophyll volume to total mesophyll volume
S:P	Spongy mesophyll volume to palisade mesophyll volume
*L*_leaf_ (µm)	Leaf thickness
LMA (mg cm^−2^)	Leaf mass per area

To explore the complex links between leaf anatomical characteristics and functional diversity in biochemical activity and CO_2_ diffusion for evergreen versus deciduous oak species, we used micro-computed tomography (μCT) to thoroughly quantify three-dimensional leaf structure within the mesophyll (e.g. mesophyll surface area, porosity, palisade mesophyll volume, and spongy mesophyll volume) (Earles et al. 2018, 2019, Lundgren and Fleming 2020, Théroux-Rancourt et al. 2020) as well as gas exchange measurements to assess photosynthetic capacity for six oak species growing in a common garden, the Peter J. Shields Oak Grove at the University of California Davis Arboretum and Public Garden (USA). These oaks were selected as pairs of sister species that differed in leaf habit, with one deciduous and one evergreen species per pair. By combining leaf biochemical and structural data with an existing time-calibrated oak phylogeny, we used a comparative physiological framework to explore whether photosynthetic capacity differed between leaf habits and to link the biochemical and anatomical characteristics of leaves to photosynthetic capacity. It is known that deciduous species exhibit a higher photosynthetic capacity than evergreen species, but the precise anatomical contributions and their mechanistic link to biochemical activity are not yet fully understood. To address this knowledge gap, we hypothesize that the higher photosynthetic capacity in deciduous leaves is a result of a coordinated strategy to maximize carbon gain during a shorter growing season. Specifically, we predict this higher capacity is driven by: (i) greater biochemical activity: a greater investment of resources into photosynthetic capacity, as evidenced by significantly higher maximum carboxylation rates (*V*_cmax_) and electron transport rates (*J*_max_) per unit leaf area and (ii) superior anatomical characteristics: a more robust and efficient internal leaf structure that facilitates gas exchange. Using μCT, we predict that deciduous leaves will have a significantly greater mesophyll surface area exposed to the IASs, which will enhance leaf internal CO_2_ diffusion. By combining detailed gas exchange measurements with quantitative anatomical analysis via μCT, our study will provide novel, mechanistic insights into how deciduous and evergreen oak species optimize their leaf structure to support their distinct photosynthetic strategies.

## Materials and methods

### Study site and species

We studied oaks (genus *Quercus*) as they make up an ecologically important model genus that dominates plant diversity and abundance in North America, Mesoamerica, and Eurasia (Nixon 1997, Hipp et al. 2018, Cavender-Bares 2019). Oak diversity is concentrated in the Americas as it is home to 260 of the roughly 400 species of oaks found globally (Nixon 1997, Hipp et al. 2014, Cavender-Bares 2019). Six oak species were selected for study in June 2022 at the Peter J. Shields Oak Grove of the University of California Davis Arboretum and Public Garden (Davis, CA, USA). We sampled three trees per oak species, except in a few cases where less than three trees were present for a given species and we re-measured individual trees to ensure a balanced experimental design as noted in the Supplementary Table S1. Collection and sampling information for the trees are also provided in Supplementary Table S1.

**Figure 1.**
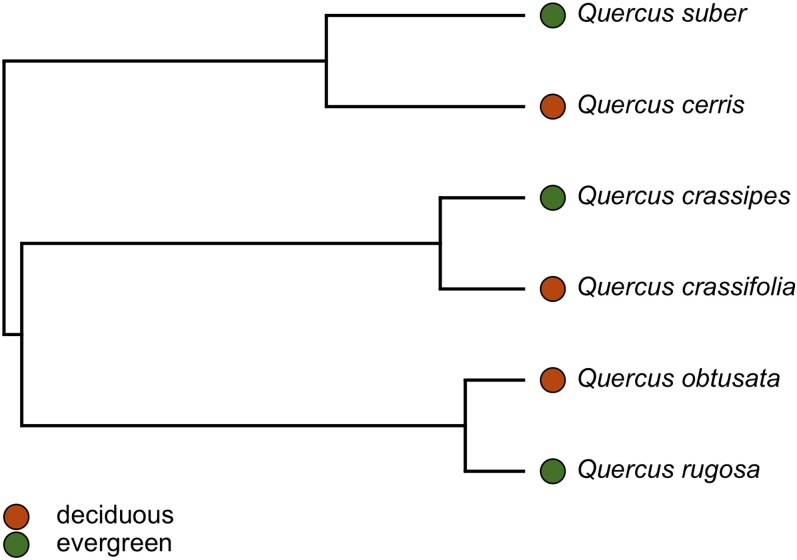
Phylogeny of oak species sampled in this study (pruned from the time-calibrated phylogeny of Hipp et al. 2020). Colour denotes the leaf habit for each species. Species and collection information are provided in the Supplementary Table S1.

To compare the biochemical and anatomical characteristics of leaves between leaf habits, the six oak species comprised three pairs of sister species that differed in leaf habit, with one deciduous and one evergreen species per pair. Using the time-calibrated phylogeny of the world’s oaks from Hipp et al. (2020) which was constructed using fossil data and restriction-site associated DNA sequencing for nearly 250 oak species, we pruned the phylogenetic tree to include our six study species (Fig. 1). Leaf habit information for each species was determined from previous studies and resources (Nixon 1997, de Beaulieu and Lamant 2010, Schmerler et al. 2012, Hipp et al. 2018, Furze et al. 2021). Deciduous species were defined by synchronous drop of leaves and their absence remained for a portion of the year, whereas evergreen species had leaves present year-round with life span >1 year (Hipp et al. 2018). Previous work suggests the leaf life span for *Q. suber* and *Quercus rugosa* is c. 1–2 years, whereas the general leaf life span across evergreen *Quercus* species is c. 1–4 years (Mediavilla and Escudero 2003, Cavender-Bares et al. 2005, Mediavilla et al. 2008, Alonso-Forn et al. 2020).

### Biochemical activity and diffusion of leaves

#### Photosynthetic measurements

Net assimilation rate (*A*_n_), stomatal conductance to CO_2_ (*g*_s_), and the IAS CO_2_ concentration (*C*_i_) were measured on one leaf from each tree using a LI-COR 6800 system fitted with a 6800-01A fluorometer. The fourth or fifth leaf was counted down from a sun-exposed branch tip, equivalent to the youngest fully developed leaf consistently at the middle canopy from the south-facing side. All measurements were conducted between 10:30 a.m. and 1:30 p.m. under photosynthetic photon flux density (PPFD) of 1500 µmol m^−2^ s^−1^ (10% blue vs. 90% red; a saturating PPFD for all species), ambient CO_2_ concentration (*C*_a_) at various levels for CO_2_ response curves (*A*_n_–*C*_i_; µmol mol^−1^), flow rate at 500 µmol air s^−1^, and vapour pressure deficit between 1.5 and 2.0 kPa. Chamber temperature was maintained at 30°C, approximating the average temperature during the photosynthetic measurement period in June for Davis, California (CIMIS 2022). To minimize potential LI-COR instrument effects, measurements were rotated and randomized across species. Stomatal conductance (*g*_s_) was measured using LI-COR 6800 at saturating PPFD of 1500 µmol m^−2^ s^−1^ prior to experimental measurements to select leaves with an active gas exchange response across trees. The LI-COR 6800 6 cm^2^ round fluorometer gasket set was filled fully with the leaf area, along with running corrections suggested by the manufacturer to reduce errors of CO_2_ and H_2_O leakage through the gasket.

#### *A*_n_−*C*_i_ curves

To better understand photosynthetic responses and estimate biochemical parameters, we constructed CO_2_ response (*A*_n_−*C*_i_) curves for each species using three replications at saturating PPFD (1500 µmol m^−2^ s^−1^) under the following sample CO_2_ concentrations: 50, 100, 150, 250, 400, 600, 800, 1200, and 1600 µmol mol^−1^ in June 2022. To validate reproducibility and account for low sample sizes in some species, *A*_n−_*C*_i_ curve measurements and derived biochemical parameters were fully replicated in June 2024, expanding the CO_2_ concentration range to include 200, 1000, 1400, and 1800 µmol mol^−1^ (Supplementary Fig. S1). Before running *A*_n_−*C*_i_ curves, the seal of the fluorometer chamber was tested for CO_2_ leakage by running a full curve at different *C*_a_ concentrations in the empty cuvette, and the measurements were corrected using the LI-COR’s equation (Bernacchi et al. 2002, Flexas et al. 2007). For each year (2022 and 2024), the *A*_n_ and corresponding *C*_i_ values for each species with three replications were initially introduced to Sharkey’s fitting calculator version 2.0 (Sharkey 2016), a Farquhar–von Caemmerer–Berry (FvCB)–based model, to estimate *V*_cmax_ and *J*_max_ (named as *J* in the Sharkey calculator as well as to predict assimilation rates (*A*_n_) at the limiting states of Rubisco (*A*_c_), RuBP regeneration (*A*_j_), and TPU (*A*_t_) (Sharkey et al. 2007) (Supplementary Figs S2 and S3). To improve the reliability and precision of derived biochemical parameters, particularly in cases where initial model fitting was suboptimal, we reanalysed *A*_n_−*C*_i_ curves using the ‘Plantecophys’ R package (Duursma 2015). For this analysis, mesophyll conductance (*g*_m_) calculated from the chlorophyll fluorescence method, as described in next section, was used as input to the FvCB model for fitting other parameters. This involved inputting a leaf temperature of 30°C, a combined Michaelis–Menten constant for CO_2_ and O_2_ of 1093.6 (μmol mol^−1^), and a Rubisco specificity of 54.9 (dimensionless) (Duursma 2015). This package, which also implements the FvCB model, yielded more consistent estimates for *V*_cmax_, *J*_max_, light respiration (*R*_d_), TPU, *A*_c_, *A*_j_ (limitation due to nicotinamide adenine dinucleotide phosphate; Medlyn et al. 2002) and *A*_p_ (analogous to *A*_t_ in Sharkey's calculator) (Supplementary Tables S3 and S6). Therefore, we selected the ‘Plantecophys’ output for presenting these parameters herein and the chlorophyll fluorescence method, as described in the next section, to estimate *g*_m_. *A*_p_ was calculated using the relationship *A*_p_ = 3TPU−*R*_d_ (Sharkey 1985, Moualeu-Ngangue et al. 2017, Busch et al. 2024), exclusively for *A*_n_−*C*_i_ curves where the ‘Plantecophys’ package confirmed a distinct steady-state for *A*_n_ limited by TPU. When TPU limitation was evident (Supplementary Table S3), the highest *A*_n_ was used as an estimate of the maximum assimilation rate (*A*_max_) achievable under non-CO_2_-limiting conditions (Sharkey et al. 2007). Further, we assessed the consistency of biochemical parameters derived from *A*_n_−*C*_i_ curves across 2022 and 2024 (Supplementary Fig. S5), and the agreement between parameter estimates generated by the Sharkey and Plantecophys models (Supplementary Fig. S6) as detailed below in the Statistical analyses section. To compare the internal photosynthetic efficiency and structural adaptations of the two leaf habits, we also calculated *A*_max_ normalized by both leaf thickness (*A*_max_/*L*_leaf_) and leaf mass (*A*_max_/LMA) (Supplementary Table S3).

#### Leaf mesophyll conductance

We chose to use a chlorophyll fluorescence method, the variable *J* method, an independent method from *A*_n_−*C*_i_ curves, to estimate *g*_m_ at CO_2_ 400 µmol mol^−1^. This method is based on calculation of electron transport rate (*J*_flu_) from measurements of chlorophyll fluorescence (Bongi and Loreto 1989, Harley et al. 1992):


(1) $$J_{\mathrm{flu}}=\Phi_{\mathrm{PSII}}\times \mathrm{PPFD}\times \alpha \times \beta$$

where β (=0.5 for C_3_ plants) is the fraction of absorbed quanta reaching photosystem II (Bernacchi et al. 2002). The leaf absorbance, α, was estimated to be 86.3% and 84.4% [±1.05% and 1.0% standard error (SE)] for deciduous and evergreen species, respectively by measuring reflectance and transmittance at full wavelength in all individuals using a portable analytical spectral device (ASD) QualitySpec Trek spectrometer (Longmont, Colorado, USA). Leaves were dark adapted for 20 min prior to all other measurements to obtain the maximum quantum yield of photosystem II. The quantum yield of photosystem II (Φ_PSII_) under actinic light was obtained by application of saturating multiphase flashes (>8000 µmol m^−2^ s^−1^) as per Genty et al. (1989) using a LI-COR 6800 system fitted with a 6800-01A fluorometer. *g*_m_ was given by Harley et al. (1992):


(2) $$g_{m}=A_{n}/\left[C_{i}-\frac{\Gamma^{*}(J_{\mathrm{flu}}+\;8(A_{n}+R_{d}))}{J_{\mathrm{flu}}-\;4(A_{n}+R_{d})}\right]$$

where Γ* is the chloroplast CO_2_ photocompensation point. The CO_2_ photocompensation point (Γ*) was assumed to be equivalent to the intercellular CO_2_ photocompensation point (*C*_i_*), as described by Gilbert et al. (2012). Using the method of Brooks and Farquhar (1985), which was adapted from Laisk (1977), we estimated *R*_d_ and *C*_i_* from the linear portion (*C*_i_ ≤ 150 µmol mol^−1^) of averaged *A*_n_−*C*_i_ curves. These curves were generated from the 2022 dataset, averaging nine sets of data for each evergreen and deciduous species group, corresponding to sample CO_2_ concentrations of 50, 100, 150, and 250 µmol mol^−1^ (Supplementary Fig. S4). The resulting *R*_d_ values were −0.88 ± 0.20 and −0.71 ± 0.20 µmol m^−2^ s^−1^, and the *C*_i_* values were 53.51 ± 3.16 and 49.60 ± 3.20 µmol mol^−1^ for deciduous and evergreen groups, respectively.

### Anatomical characteristics of leaves

#### Micro-computed tomography imaging

**Figure 2.**
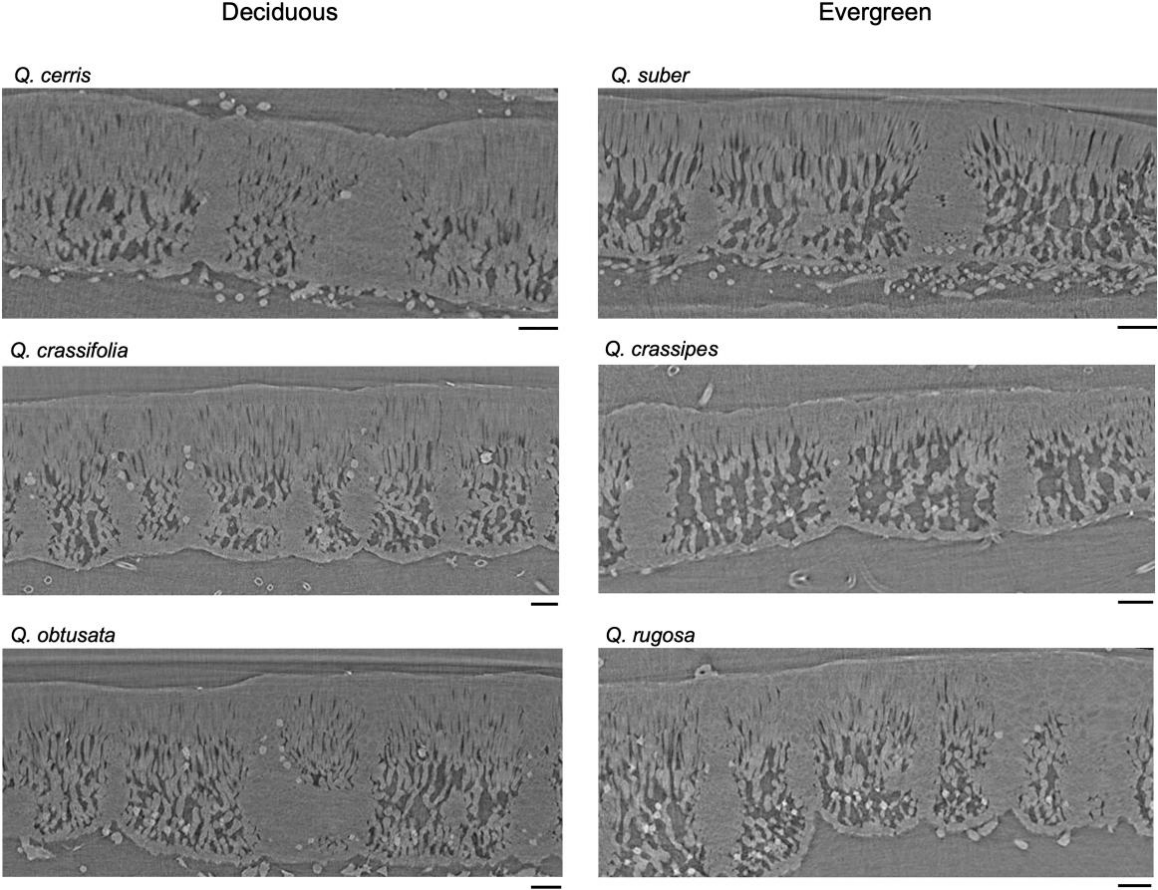
Leaf cross sections from representative μCT images for each deciduous (left column) and evergreen (right column) *Quercus* species from 2022. Each row contains a species pair based on the phylogeny in Fig. 1. Each scale bar = 50 µm.

Leaves were scanned using μCT imaging at beamline 8.3.2 at the Advanced Light Source (ALS) in Lawrence Berkeley National Laboratory, Berkeley, CA, USA (Fig. 2). The same leaf samples used for gas exchange were collected from the trees, bagged, and placed in a cooler at room temperature an hour before scanning at the ALS. A single rectangle (c.a., 3 mm wide × 7 mm long) was cut from the middle of each leaf lamina using a razor blade and enclosed between two pieces of Kapton tape to prevent tissue desiccation and sample movement during the scanning. Samples were placed inside the end of a pipette tip and scanned under a continuous tomography mode at 23 keV using a 10× objective lens with a pixel resolution of 0.65 μm. Raw tomographic data were reconstructed using TomoPy (Gürsoy et al. 2014) through gridrec reconstruction (Davis et al. 1995, Dowd et al. 1999).

#### Segmentation and fully convolutional network model

Consecutive slices from each grid stack (*n* = 530 slices total) were selected for manual segmentation using Image J software (v. 2.9.0, US National Institutes of Health, Bethesda, MD, USA) and a Wacom tablet (2023 Intuos Pro Medium, Wacom Technology Corporation, Portland, OR, USA). The resulting image stack was segmented using the methods presented by Momayyezi et al. (2022b). Six slices were manually masked for various leaf tissues per scan (1 leaf scan per tree; 18 scans total). The manually segmented slices had individual labels for the adaxial epidermis, abaxial epidermis, palisade mesophyll cells, spongy mesophyll cells, IAS, bundle sheath extensions, veins, and background outside of the scanned leaf. Masks and associated images from 3 trees per species (*n* = 18 masks total) were pulled together to run a ‘big model’ using a fully convolutional network (FCN) model. Twelve and six masks and images were used for training and testing FCN, respectively. A PyTorch implementation of FCN model with a ResNet-101 backbone was used for the semantic segmentation of the leaf image data with cloud-based resources in Google Colab. Ten training models were evaluated to select the optimal model based on F1 score. For epidermis and mesophyll tissues, prediction F1 scores were >80% while prediction F1 scores were generally >75% for airspaces and >70% for bundle sheath extensions and veins (Supplementary Table S2). These results aligned with those reported by Rippner et al. (2022) for the FCN model and were comparable to the performance achieved by Théroux-Rancourt et al. (2020) using a Random Forest method. For training, we used a binary cross-entropy loss function, an Adam optimizer for stochastic optimization with a learning rate of 0.001, a scaling factor of 1 to avoid small feature loss in the training images, and a batch size of 1 to accommodate the graphics processing unit limitations in Google Colab.

#### Mesophyll surface area, mesophyll porosity, palisade mesophyll and spongy mesophyll volume, and leaf thickness

The surface area of the whole mesophyll [*SA*_mes(S+P)_] and for each palisade [*SA*_mes(P)_] and spongy [*SA*_mes(S)_] were calculated as the ratio of spongy mesophyll and palisade mesophyll surface area [*SA*_mes(S+P)_] to the whole mesophyll volume (*V*_mes_: *V*_P_ + *V*_S_ + θ_IAS_) as described by Momayyezi et al. (2022b) and in Table 1. Mesophyll porosity (θ_IAS_; m^3^ m^−3^) was calculated as the IAS volume as a fraction of the total mesophyll volume. The palisade mesophyll and spongy mesophyll cell volume to total mesophyll volume ratios were calculated as P:M (the ratio of palisade volume to total mesophyll volume) and S:M (the ratio of spongy volume to total mesophyll volume), respectively. The spongy to palisade mesophyll volume ratio was calculated and presented as S:P. Leaf thickness (*L*_leaf_) was calculated on the μCT images using the line tool in Image J with five lines per image and using six images per individual (the same six images that were manually segmented by leaf tissue).

### Biochemical, diffusional, and anatomical parameters

Above we have described the measurement of biochemical, diffusional, anatomical, and morphological characteristics of leaves. A summary table of the abbreviations used for these parameters and their definitions are provided in Table 1. Since μCT images of leaves were obtained in 2022, we herein present biochemical, diffusional, and anatomical parameters from 2022 only. All biochemical and diffusional data from 2024 are presented in the Supplementary material.

### Statistical analyses

Data for biochemical, CO_2_ diffusional, anatomical, and morphological characteristics of leaves for each tree for 2022 and 2024 are provided in Supplementary Table S3. Biochemical, CO_2_ diffusional and anatomical characteristics of leaves from 2022 were compared between leaf habits (Supplementary Table S4). The biochemical parameters were *V*_cmax_, *J*_max_, TPU, *A*_max_, *A*_n_ (at 400 µmol mol^−1^), and *R*_d_; CO_2_ diffusional parameters were *g*_s_ and *g*_m_ (at 400 µmol mol^−1^); the anatomical characteristics were *SA*_mes(S+P)_, *SA*_mes(P)_, *SA*_mes(S)_, θ_IAS_, P:M, S:M, and S:P, while *L*_leaf_ and LMA were leaf morphological characteristics. Data were then averaged to obtain species’ means for each trait to perform analyses in a phylogenetic context. To statistically test whether each trait differed between leaf habits, we used the ‘procD.pgls’ function in the R package, *geomorph* version 4.0.5 (R Core Team 2022, Adams et al. 2025) to perform phylogenetic analysis of variance [phylogenetic analysis of variance (ANOVA)] based on 1000 permutations (Supplementary Table S4).

Furthermore, linear regression was used to examine the relationships between *A*_n_ and each biochemical and anatomical parameter (Supplementary Table S5). Relationships between *A*_max_ and each biochemical and anatomical parameter were also assessed (Supplementary Fig. S7). Strength of association was evaluated using Pearson’s correlation at α = 0.05.

We also conducted linear regression and Pearson correlation analyses to evaluate two key aspects: (i) the consistency of biochemical parameters derived from *A*_n_−*C*_i_ curves across 2022 and 2024 (Supplementary Fig. S5) and (ii) the agreement between parameter estimates generated by the Sharkey and Plantecophys models (Supplementary Fig. S6). Linear regression was employed to quantify differences between the model outputs, and Pearson correlation was used to assess the strength of the relationship between the two methods.

## Results

### Deciduous species have higher photosynthetic capacity than evergreen species

Photosynthetic capacity, as measured by *A*_max_, was significantly higher for deciduous species (23.18 ± 8.53) than evergreen species (12.37 ± 3.04) (Fig. 4; Supplementary Table S4). *A*_max_ (thickness) was also significantly higher for deciduous species (0.10 ± 0.01 vs. 0.06 ± 0.01) while there was no significant difference between the two leaf habits in *A*_max_ (mass) (Supplementary Table S3). Biochemical and anatomical characteristics of leaves were also compared between leaf habits to assess their relationship to the higher photosynthetic capacity of deciduous species. Biochemical parameters (i.e. *V*_cmax_, *J*_max_, and TPU) and anatomical characteristics [i.e. P:M and *SA*_mes(S+P)_] were significantly higher for deciduous species compared to evergreen (Fig. 4; Supplementary Table S4). S:P was significantly lower for deciduous leaves (0.59 ± 0.13) compared to evergreen (0.90 ± 0.28) (Fig. 4; Supplementary Table S4). θ_IAS_ was also significantly lower for deciduous leaves (0.23 ± 0.07) compared to evergreen (0.32 ± 0.05) (Fig. 4; Supplementary Table S4).

Leaf biochemical, diffusional, and anatomical characteristics were then assessed for their association with higher photosynthetic capacity. Higher *A*_max_ was associated with higher *V*_cmax_, *J*_max_, *g*_s,_  *g*_m_, and P:M, but lower θ_IAS_ (Supplementary Fig. S7). Similar to *A*_max_, higher *A*_n_ was also associated with higher *V*_cmax_, *J*_max_, and *g*_s_ (Fig. 5; Supplementary Table S5). *A*_n_ was not directly associated with any anatomical or morphological characteristics (Supplementary Fig. S8 and Table S5). Deciduous species tended to show higher *g*_m_ than evergreen species, but the difference was not significantly different (Supplementary Fig. S9).

### Leaf anatomical characteristics facilitate higher photosynthetic capacity in deciduous species

In our study, deciduous species with significantly higher P:M (Fig. 4b) and significantly lower S:P (Fig. 4b) tended to have higher *A*_n_ and *g*_m_ at 400 µmol mol^−1^ ambient CO_2_ (Supplementary Fig. S9). Leaf morphological traits, *L*_leaf_ and LMA, did not significantly differ between leaf habits (Supplementary Fig. S9 and Table S5). Deciduous leaves exhibited significantly higher P:M and lower S:P and θ_IAS_, with *SA*_mes(S+P)_ also showing a borderline significant increase (Fig. 4b; Supplementary Table S4). Collectively, these traits suggest a strategy of investing in a greater mesophyll surface area supported by a densely packed palisade mesophyll structure. This contrasts with evergreens, where the functional effect of a lower palisade proportion (P:M) may be partially compensated for by higher θ_IAS_, thereby balancing the overall mesophyll surface area ratio.

## Discussion

Oak species with different leaf habits and living in a common garden setting exhibited variable photosynthetic capacity. Photosynthetic capacity was significantly higher in deciduous species compared to evergreen (Fig. 4; Supplementary Table S4), which is in agreement with our hypothesis. High photosynthetic capacity was related to carboxylation capacity, which is often due in part to the high activity and abundance of the Rubisco enzyme (von Caemmerer and Farquhar 1981, Hikosaka and Shigeno 2009, Kattge et al. 2011). As expected, deciduous species tended to have higher *A*_n_ and *g*_m_ (at 400 µmol mol^−1^) and significantly higher *A*_max_. Both *A*_max_ and *A*_n_ tended to be significantly and positively correlated with *V*_cmax_, *J*_max_, and *g*_s_, and *g*_m_, further corroborating the link between high biochemical activity, CO_2_ diffusion, and high leaf-level photosynthetic performance. Leaf anatomical characteristics were different between the two leaf habits and some of them were significantly associated with *A*_max_.

**Figure 3.**
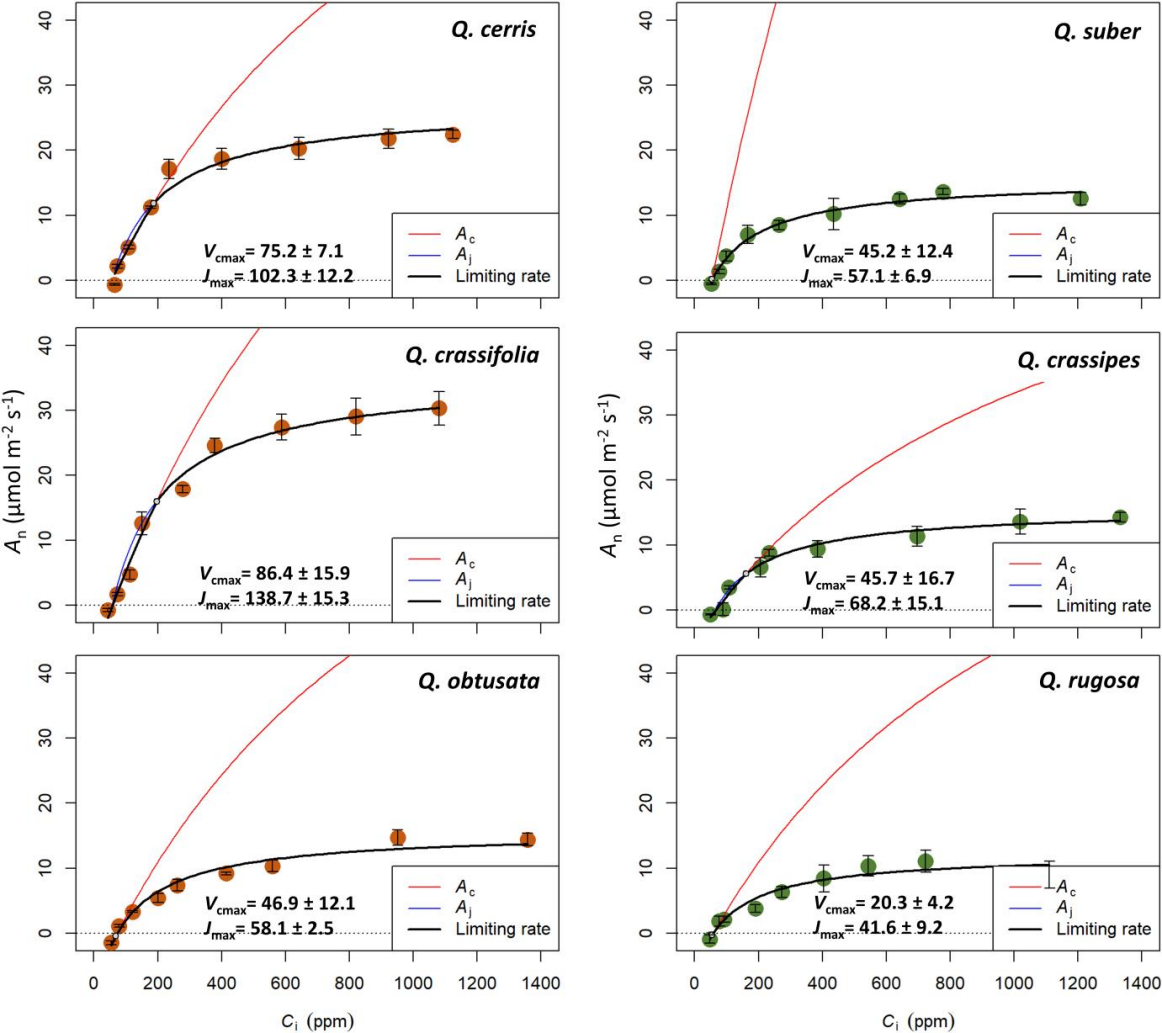
Photosynthetic CO_2_ response curves were constructed using plantecophys package (Duursma 2015), averaged over three replications for six *Quercus* species from 2022. *A*_n_ − *C*_i_ curves are shown for deciduous species (left column) and evergreen species (right column), and error bars from direct measurements. *A*_n_−*C*_i_ curves were used to generate the maximum carboxylation rate (*V*_cmax_) and the maximum electron transport rate (*J*_max_) and averaged over three measurements for each species in 2022 (±SE, *n* = 3). The Rubisco and RuBP regeneration limitations are indicated for each accession by red and blue curves, respectively.

**Figure 4.**
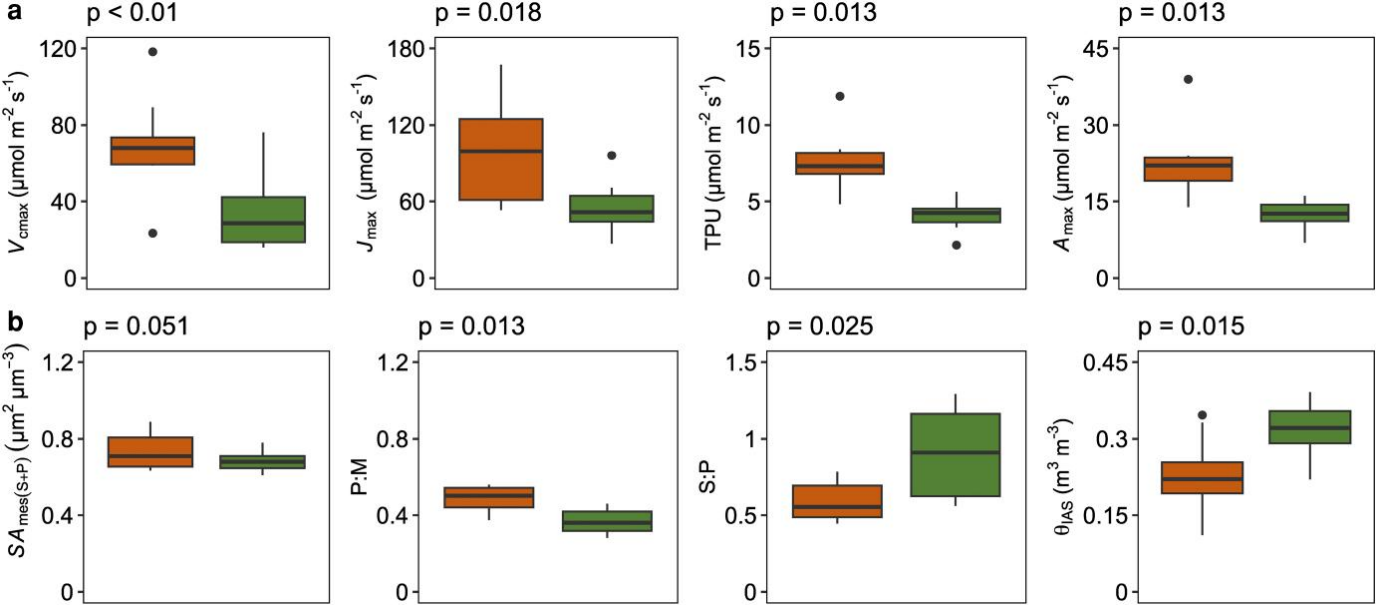
Comparison of (a) biochemical and (b) anatomical characteristics of leaves between leaf habits from 2022. Colour indicates leaf habit (deciduous = orange, evergreen = green; deciduous is the left boxplot and evergreen is the right boxplot within each graph). *P*-values displayed are from phylogenetic ANOVA testing based on 1000 residual randomization permutations. Summary output from phylogenetic ANOVA testing is provided in Supplementary Table S4. Biochemical and anatomical characteristics of leaves that were not significantly different between leaf habits are displayed in Supplementary Figure S9. In each boxplot, the box represents the interquartile range spanning from the 25th to 75th percentiles, the horizontal line within the box represents the median, whiskers extend to the minimum and maximum values, and points are outliers. The analysis includes a total of nine measurements (*n* = 9) for each evergreen and deciduous species group.

Overall, our results support the idea that the biochemical activity and anatomical characteristics of leaves contribute to the difference in photosynthetic capacity between leaf habits, with deciduous species exhibiting higher *V*_cmax_, *J*_max_, *g*_m_, *SA*_mes(S+P)_, P:M, and lower θ_IAS_ and S:P compared to evergreen species (Fig. 4). Deciduous species showed higher performance through increasing *V*_cmax_, as estimated by the net assimilation rate response to *C*_i_ (*A*_n_−*C*_i_ curve), when Rubisco becomes limiting to *A*_n_ (i.e. *A*_c_) (Fig. 3). That finding was in line with more Rubisco activity (Warren and Adams 2004, Sharkey et al. 2007, Niinemets et al. 2009b) which enabled them to increase their *A*_n_ at lower CO_2_ concentrations (Fig. 3). Greater *J*_max_, where RuBP regeneration limits *A*_n_ (i.e. *A*_j_), was supported by the combined effects of their significantly lower θ_IAS_ and higher P:M ratio (Fig. 4). This combination of dense cell packing is also reflected in the increased, though borderline significant, *SA*_mes(S+P)_, which represents the total mesophyll surface area. Specifically, mesophyll surface area per unit leaf area is strongly driven by anatomical traits such as greater leaf thickness and lower porosity, though the mesophyll cell surface to volume ratio can exert a balancing effect, leading to a smaller overall mesophyll surface area (as reported in sun-induced differences by Théroux-Rancourt et al. 2023). *A*_max_ was strongly correlated with *V*_cmax_ and *J*_max_ (Supplementary Fig. S7), indicating that deciduous leaves possess a greater biochemical capacity. This can be attributed to their densely packed mesophyll, which includes a larger volume of palisade tissue, providing more surface area for enzymatic activity and photosynthetic processes. Greater TPU, also estimated by *A*_n_−*C*_i_ curves for deciduous species, showed less limitation to *A*_n_ for these species. They had a greater capacity to fix carbon than remove it from the Calvin–Benson cycle (McClain and Sharkey 2019), which was in agreement with their high starch accumulation reported previously (Furze et al. 2021).

Even though leaf thickness was similar between leaf habits, the palisade mesophyll volume was larger in deciduous species compared to evergreen, increasing the photosynthesizing surface area for biochemical activities and light capture. The palisade mesophyll facilitates uniform distribution of light within the leaf due to its columnar cell shape, unlike spherical-shaped cells of the spongy mesophyll which help light scattering due to more IAS (Knapp et al. 1988, Vogelmann 1993, Evans 1999). In addition, columnar cells of the palisade mesophyll provide optimum vertical geometry to maximize the number of chloroplasts exposed to the IAS (Niinemets et al. 2009a, Terashima et al. 2011, Ren et al. 2019). Thus, more mesophyll surface area exposed to IAS may facilitate CO_2_ diffusion (i.e. greater *g*_m_) and light absorption, both contributing to high photosynthesis and providing deciduous species with a photosynthetic advantage (Vogelmann 1993, Terashima and Hikosaka 1995, Smith et al. 1997, Johnson et al. 2005, Kume 2017, Gotoh et al. 2018). This agrees with our findings for deciduous oaks with higher *SA*_mes(S+P)_ and tended to have higher *g*_m_. In addition, the P:M contribution to higher *A*_max_ highlights the significance of palisade mesophyll in improving light absorption and CO_2_ diffusion for deciduous species in this study. θ_IAS_ was lower for deciduous species, meaning that the mesophyll tissue was more densely packed than in evergreens. This aligns with previous findings, such as those in grapevine, suggesting that high porosity leads to lower photosynthetic capacity on an area basis (Théroux-Rancourt et al. 2023). However, this result contrasts with previous work in walnuts—which are deciduous species—showing that higher θ_IAS_ (i.e. more porous mesophyll with lower cell density) can enhance photosynthetic capacity by improving gas phase diffusion inside mesophyll tissue (Momayyezi et al. 2022b). This aligns with the general observation that more porous leaves exhibit lower diffusive resistance in the gas phase due to shorter path lengthening (Earles et al. 2018, Momayyezi et al. 2022a). Nevertheless, θ_IAS_ is not the only anatomical factor that influences the diffusive resistance of CO_2_; besides the mesophyll surface area, other factors that we did not directly measure that could influence mesophyll CO_2_ diffusion via changes in the liquid phase include mesophyll cell diameter, and cell wall thickness (Flexas et al. 2008, Théroux-Rancourt and Gilbert 2017, Evans 2021, Knauer et al. 2022). Some recent studies suggest a dynamic relationship between mesophyll surface area, airspace development, and stomatal patterning which can feedback on one another for optimal gas exchange, matching the growth environment (Lundgren et al. 2019, Baillie and Fleming 2020). Reinforcing these findings, ongoing research explores increasing porosity and lowering cell wall thickness through crop engineering approaches to enhance photosynthesis (Salesse-Smith et al. 2024).

Thus, the combination of leaf biochemistry and anatomical characteristics that favour higher photosynthetic capacity in deciduous oaks illustrates a trade-off between leaf habits. Deciduous species have higher photosynthetic rates to compensate for a shorter growing season and evergreen species have lower photosynthetic rates but can accommodate this by being able to photosynthesize during favourable conditions year-round (Hollinger 1992, Baldocchi et al. 2010). The leaf anatomy of evergreen oaks, characterized by a thicker spongy mesophyll, serves as a protective mechanism against environmental stressors (Sancho-Knapik et al. 2021). This increased spongy thickness provides a crucial survival advantage by creating more IAS (greater porosity) to protect against frost damage and photodamage (Wyka and Oleksyn 2014). This highlights a key trade-off in evergreen oaks which prioritize structural investment for year-round protection over maximizing features for high photosynthetic activity, such as increased palisade density and mesophyll surface area, which are more common in deciduous oaks. Interestingly, leaf anatomical characteristics of deciduous oak species resemble those typically found in sun-exposed leaves (regardless of leaf habit), including a thicker palisade mesophyll and lower mesophyll porosity. Abundant photosynthate in sun-exposed leaves is predicted to shift leaf development towards a denser packing of cells (Yano and Terashima 2001, Terashima and Yano 2004). This is in agreement with our observations for deciduous oaks to show greater *A*_max_ on a leaf thickness basis, confirming their adaptation during the favourable season with building a more robust leaf anatomy (e.g. more nitrogen content per unit area, greater mesophyll surface area) (Givnish 2002). With higher photosynthetic rates and larger non-structural carbohydrate reserves in deciduous oaks (Furze et al. 2021), more photosynthate may also support the direction of leaf development to increase mesophyll surface area, similar to sun-exposed leaves with high carboxylation capacity (Terashima et al. 2006). More non-structural carbohydrates can also support higher vein density in sun-exposed leaves as a significant sink that facilitates sugar transport towards leaves (MacNeill et al. 2017, Théroux-Rancourt et al. 2023).

**Figure 5.**
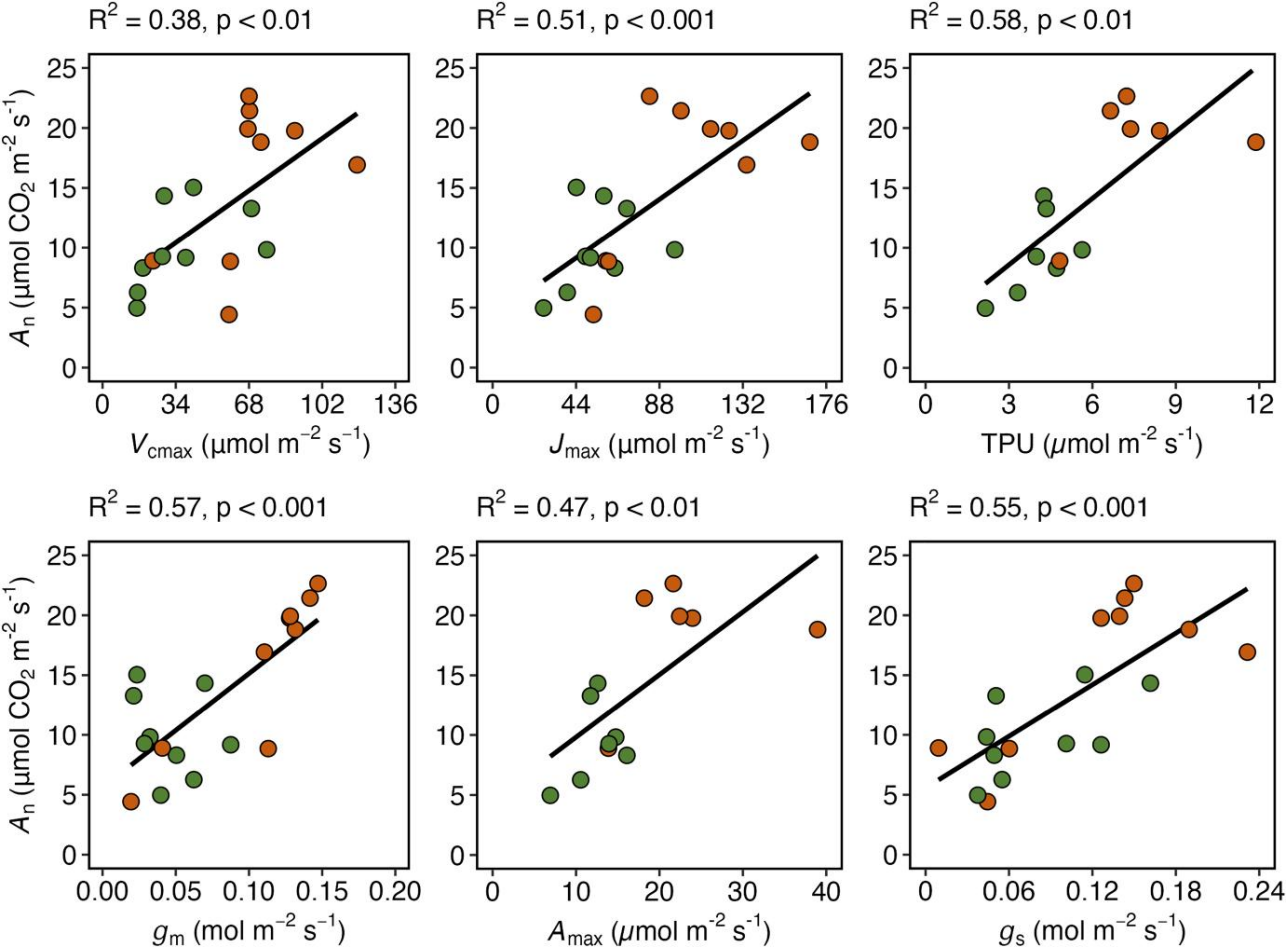
Net assimilation rate (*A*_n_) at ambient CO_2_ concentration (*C*_a_ at 400 µmol mol^−1^) relationship with diffusional (*g*_m_ and *g*_s_ at *C*_a_ at 400 µmol mol^−1^) and biochemical (*V*_cmax_, *J*_max_, TPU, and *A*_max_) leaf characteristics. Each point represents an individual tree with color indicating leaf habit (deciduous = orange, evergreen = green). *A*_n_ and *g*_s_ were extracted from *A*_n_−*C*_i_ curves at *C*_a_ of 400 µmol mol^−1^. *g*_m_ was estimated using the chlorophyll fluorescence method at *C*_a_ of 400 µmol mol^−1^. Strength of association was evaluated with Pearson’s correlation at α = 0.05. Summary output from correlation analyses is provided in Supplementary Table S5. Additional associations that were not statistically significant are displayed in Supplementary Figure S8.

Future studies could measure chlorophyll-related leaf spectra and light absorption profiles to investigate the role of pigments in driving photosynthetic capacity differences between leaf habits. Normalized pigment chlorophyll index (NPCI) is a spectral parameter used to estimate chlorophyll content and reflects the ratio of carotenoids to chlorophyll *a*, which could further inform our understanding of deciduous versus evergreen oak photosynthetic capacity. For example, carotenoids absorb light in different spectral regions than chlorophyll, thereby extending the wavelengths of light that can be used for photosynthesis. Additionally, carotenoids transfer light energy to chlorophyll and help protect the photosynthetic apparatus from photo-oxidative damage due to excess light energy (Polívka and Frank, 2010). Thus, deciduous oaks may have a higher NPCI in support of higher photosynthetic capacity. Furthermore, measuring the spatial distribution of chlorophyll across leaf cross sections—which has been studied broadly across vascular plants (Borsuk and Brodersen 2019)—could be a useful tool to further examine patterns of light absorption by chlorophyll and their link to photosynthetic capacity in deciduous and evergreen species (Fig. 5).

Here, we used a comparative physiological framework to explore differences in the biochemistry, CO_2_ diffusion, and anatomical characteristics of evergreen and deciduous leaves that influence photosynthetic capacity. Notably, we found that deciduous oak species, with more than two-fold higher *V*_cmax_ and *J*_max_, denser mesophyll packed with more palisade than spongy cells, and higher mesophyll surface area, showed higher photosynthetic capacity than evergreen oak species. Their greater palisade mesophyll volume and higher mesophyll surface area supported their capacity for greater CO_2_ diffusion, biochemical activities, and photosynthetic capacity (e.g. *A*_max_). Together, the biochemistry and anatomical characteristics of deciduous leaves support greater photosynthetic capacity to compensate for the shorter growing season they endure.

## Supplementary Material

plaf063_Supplementary_Data

## Data Availability

The data that support the findings of this study are available in the Supplementary material of this article, and any data or code beyond that can be obtained from the corresponding author upon reasonable request.
