# Supplementary material for: Biochemical and anatomical leaf characteristics of oak trees contribute to differences in photosynthetic capacity between leaf habits
Source: AoB Plants. 2025 Nov 3;17(6):plaf063. doi: 10.1093/aobpla/plaf063 (PMC12603363; doi:10.1093/aobpla/plaf063)
Supplement: plaf063_Supplementary_Data [file plaf063_supplementary_data.pdf]

Supplementary data

The following supplementary data are available for this article:

Fig. S1 Photosynthetic CO_2 response curves constructed using Plantecophys package for 2024 data

Fig S2 Photosynthetic CO_2 response curves constructed using Sharkey's fitting calculator for 2022 data

Fig S3 Photosynthetic CO_2 response curves constructed using Sharkey's fitting calculator for 2022 data

Fig S4 Estimation of light respiration and CO_2 photocompensation point using Brooks and Farquhar method for 2022

Fig S5 Comparison of biochemical parameters derived from Plantecophys models between 2022 and 2024 data

Fig S6 Comparison of biochemical parameter estimates generated by Sharkey and Plantecophys models for 2022 data

Fig S7 Maximum assimilation rate (A_{max}) relationship with biochemical, diffusional, anatomical, and morphological characteristics of leaves from 2022

Fig S8 Net assimilation rate (A_n) relationship with biochemical, anatomical, and morphological characteristics of leaves (non-significant relationships) from 2022

Fig. S9 Comparison of biochemical and anatomical characteristics of leaves between leaf habits (non-significant comparisons) from 2022

Table S1 Collection information for the individual trees in the study

Table S2. Precision, accuracy, recall, and F1 scores as a function of annotated leaves from 2022

Table S3 Biochemical, diffusional, anatomical, and morphological data for each tree from (A) 2022 and (B) 2024

Table S4 Summary output from phylogenetic ANOVAs of 2022 data

Table S5 Summary output from correlation analyses of 2022 data

Table S6 Net assimilation rate (A_n) response to intercellular airspace CO_2 concentrations (C_i), assimilation rates at Rubisco (A_c) and RuBP regeneration (A_j) limiting states conducted in 2022 and 2024

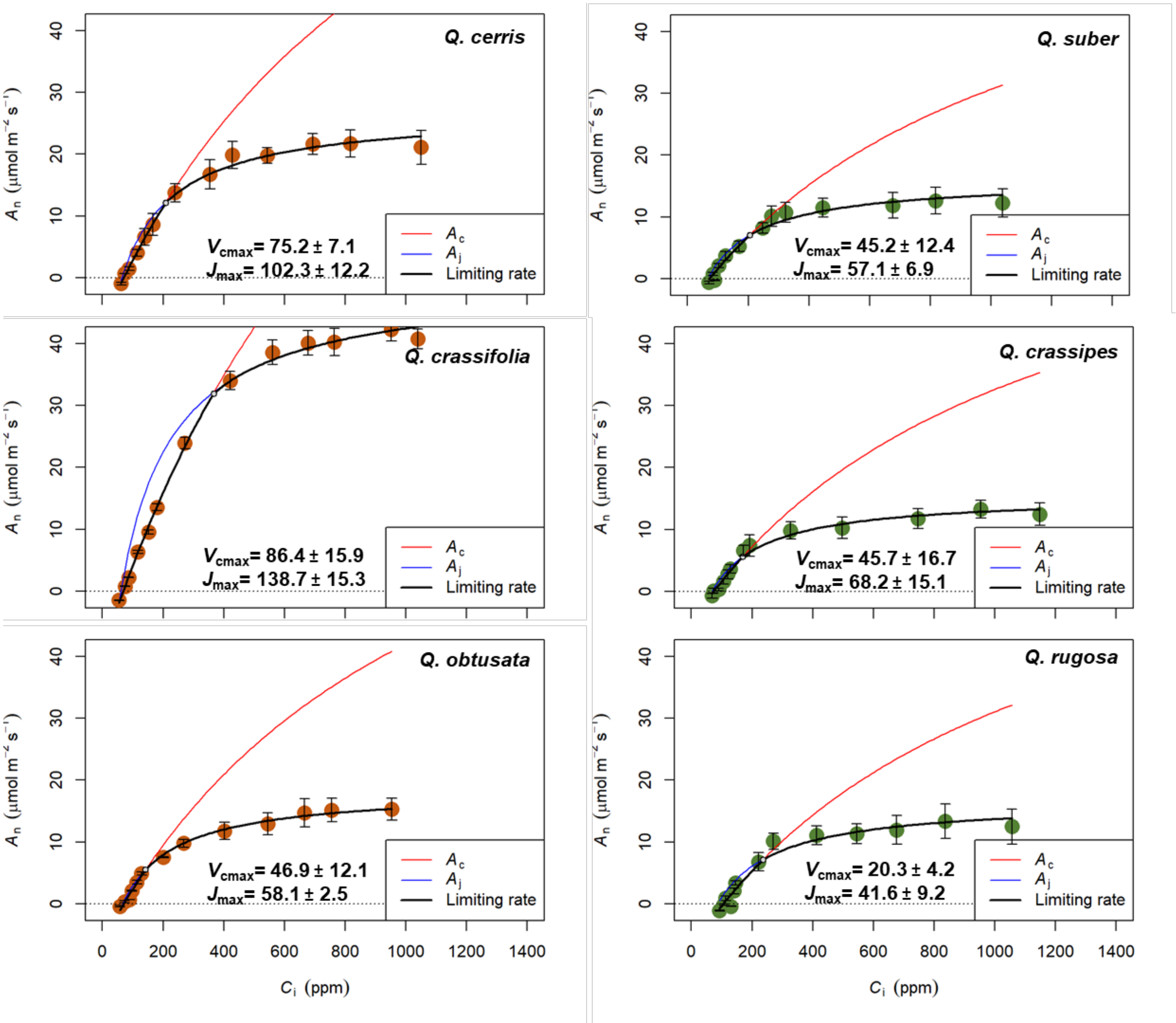

Figure S1. Photosynthetic CO_2 response curves were constructed using plantecophys package (Duursma 2015) and averaged over three replications for six *Quercus* species using 2024 curve measurements. The CO_2 concentration range for the A_n-C_i curves measured in June 2024 was expanded to 200, 1000, 1400, and 1800 μmol mol-1, compared to the narrower range used in June 2022. A_n-C_i curves were used to generate the maximum carboxylation rate (V_{cmax}) and the maximum electron transport rate (J_{max}) and averaged over three replicated measurements for each species (± SE, n = 3). Green circles represent evergreen species, while orange circles represent deciduous species.

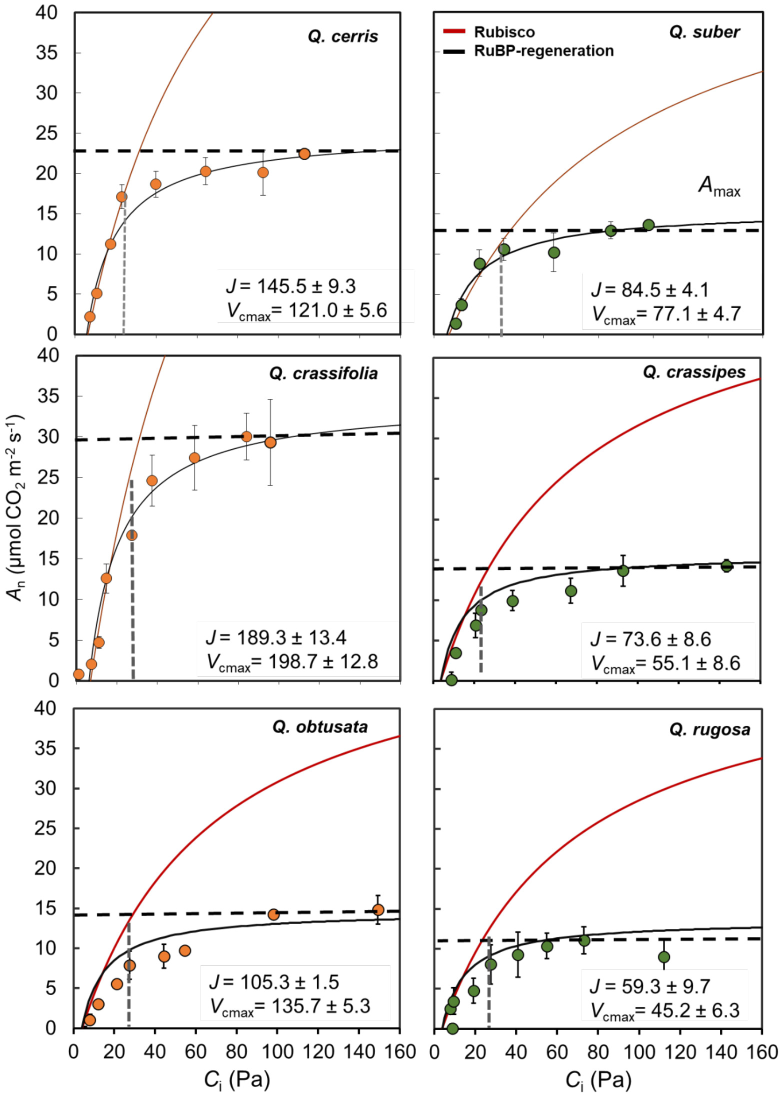

Figure S2. Photosynthetic CO_2 response curves were constructed using Sharkey's fitting calculator version 2.0 (Sharkey 2016), averaged over three replications for six *Quercus* species. These curves were collected in June 2022. A_n-C_i curves are shown with colored circles, green for evergreen and orange for deciduous species, and error bars from direct measurements. A_n-C_i curves were used to generate the maximum carboxylation rate (V_{cmax}) and the electron transport rate (J) and averaged over three replicated measurements for each species (± SE, n = 3). The black dashed horizontal line indicates the maximum assimilation rate (A_{max}) estimated where CO_2 is no longer limiting A_n (i.e., at triose phosphate use (TPU) limitation state), and Rubisco and RuBP regeneration limitations are indicated for each accession by red and black curves, respectively. Dashed vertical gray lines on each plot represent the estimated C_i at ambient CO_2 of 400 µmol mol^{-1} (~ 40.5 Pa), which represents the limitation of g_m to A_n (i.e., the point where C_i and C_c would be equal).

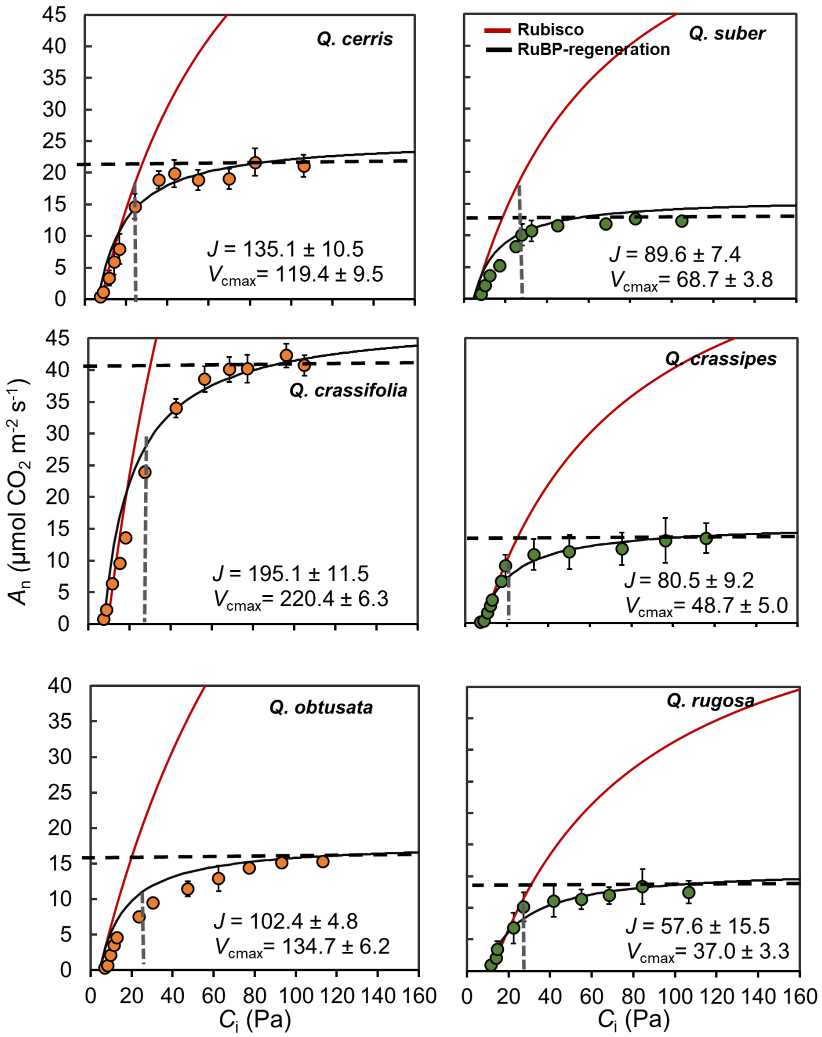

Figure S3. Photosynthetic CO_2 response curves were constructed using Sharkey's fitting calculator version 2.0 (Sharkey 2016), averaged over three replications for six *Quercus* species. These curves were collected in June 2024. A_n-C_i curves are shown with colored circles, green for evergreen and orange for deciduous species, and error bars from direct measurements. A_n-C_i curves were used to generate the maximum carboxylation rate (V_{cmax}) and the electron transport rate (J) and averaged over three replicated measurements for each species (± SE, n = 3). The black dashed horizontal line indicates the maximum assimilation rate (A_{max}) estimated where CO_2 is no longer limiting A_n (i.e., at triose phosphate use (TPU) limitation state), and Rubisco and RuBP regeneration limitations are indicated for each accession by red and black curves, respectively. Dashed vertical gray lines on each plot represent the estimated C_i at ambient CO_2 of 400 µmol mol^{-1} (~ 40.5 Pa), which represents the limitation of g_m to A_n (i.e., the point where C_i and C_c would be equal).

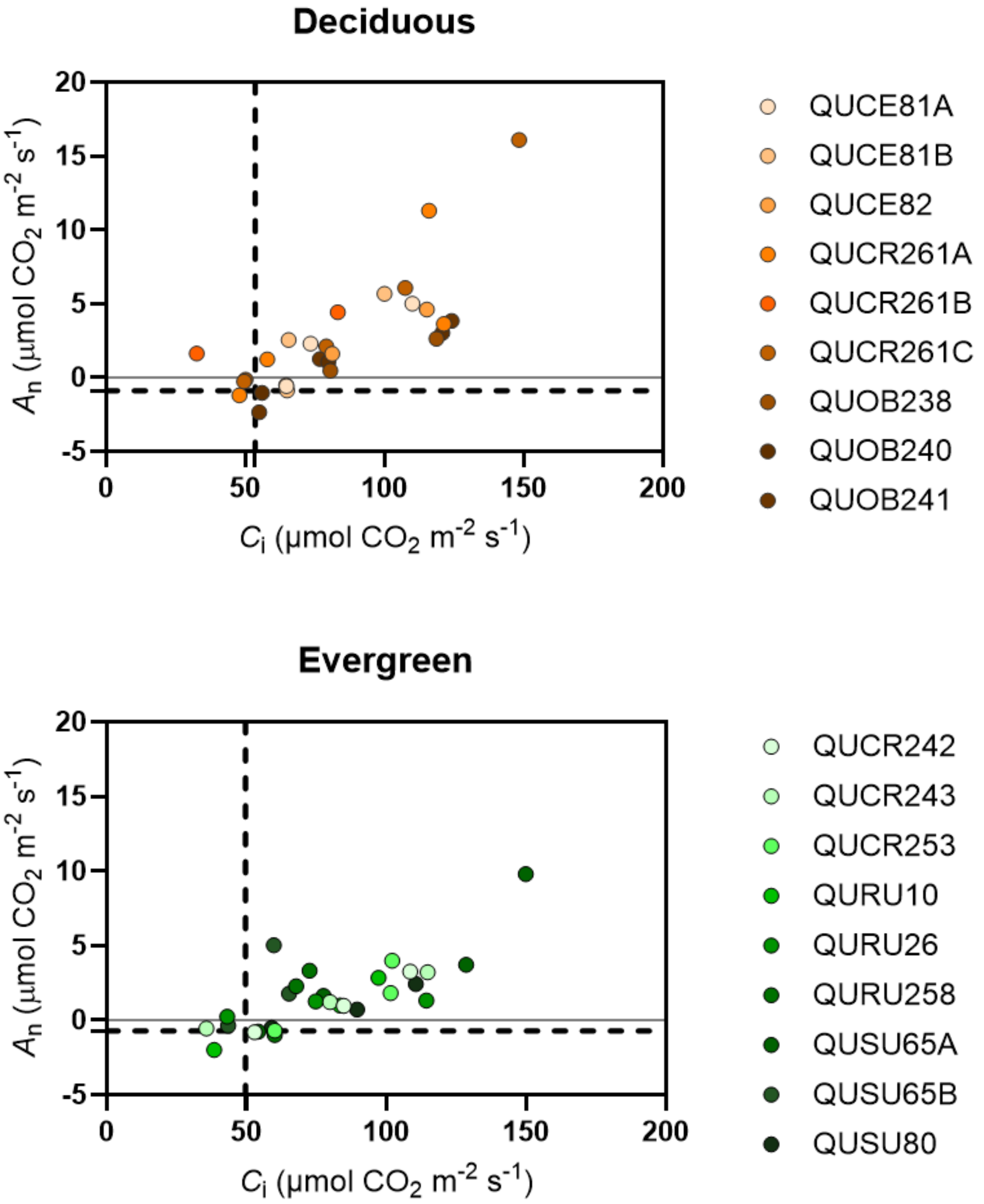

Figure S4. Estimation of respiration rate in the light (R_d) and CO_2 photocompensation point (C_i*) using Brooks & Farquhar (1985) method. The linear portion (C_i ≤ 150 µmol mol^{-1}) of A_n-C_i curves from the 2022 dataset were used to calculate the respiration rate in the light (R_d) and CO_2 photocompensation point (C_i*) for deciduous and evergreen species.

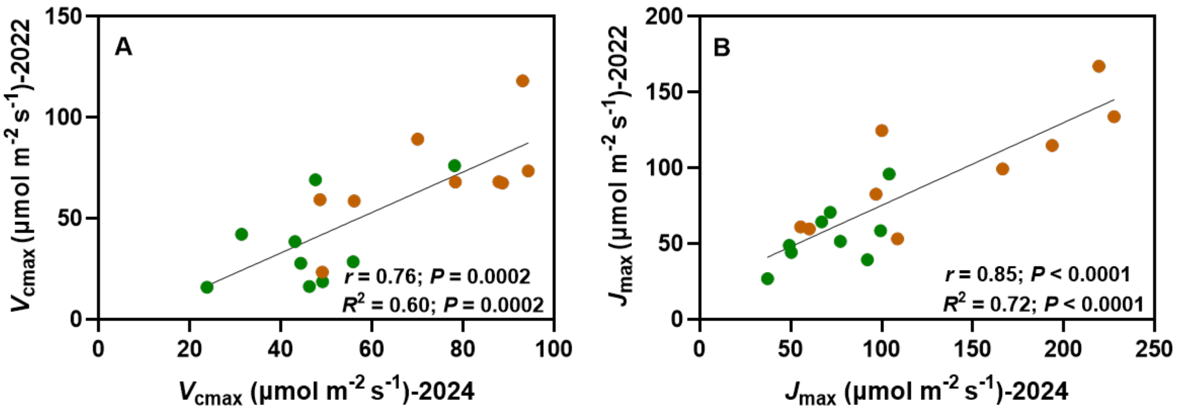

Figure S5. Linear regression and Pearson correlation comparing A) V_{cmax} and B) J_{max} estimates from 2022 and 2024 A_n-C_i curves measurements using plantecophys R package.

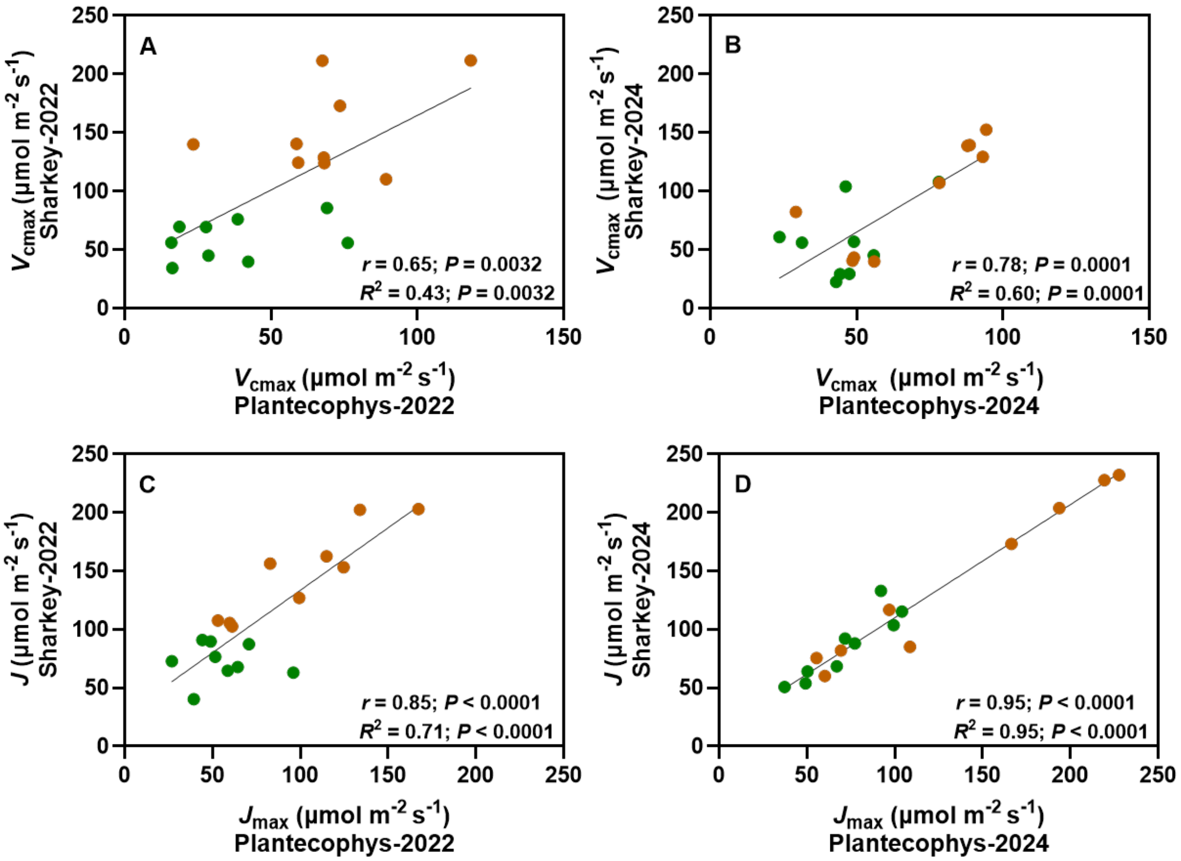

Figure S6. Linear regression and Pearson correlation comparing A-B) V_{cmax} vs. V_{cmax} and C-D) J_{max} vs. J estimates from 2022 and 2024 A_n-C_i curves using plantecophys R package and Sharkey's fitting calculator version 2.0 (Sharkey 2016).

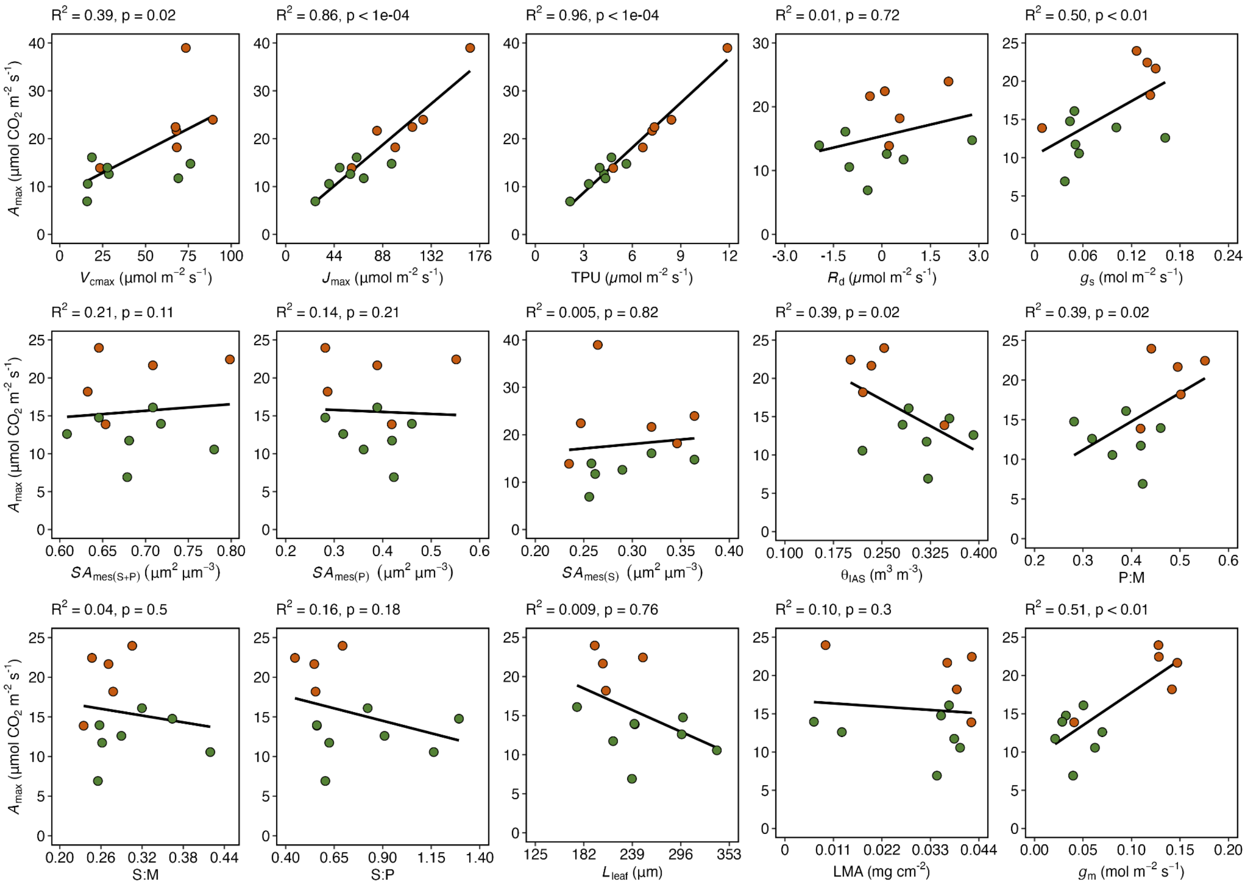

Figure S7. $A_{\max}$ relationship with biochemical and anatomical characteristics of leaves from 2022. Each point represents an individual tree with color indicating leaf habit (deciduous=orange, evergreen=green). Strength of association was evaluated with Pearson's correlation at α = 0.05. Abbreviations along x-axes are defined in Table 1.

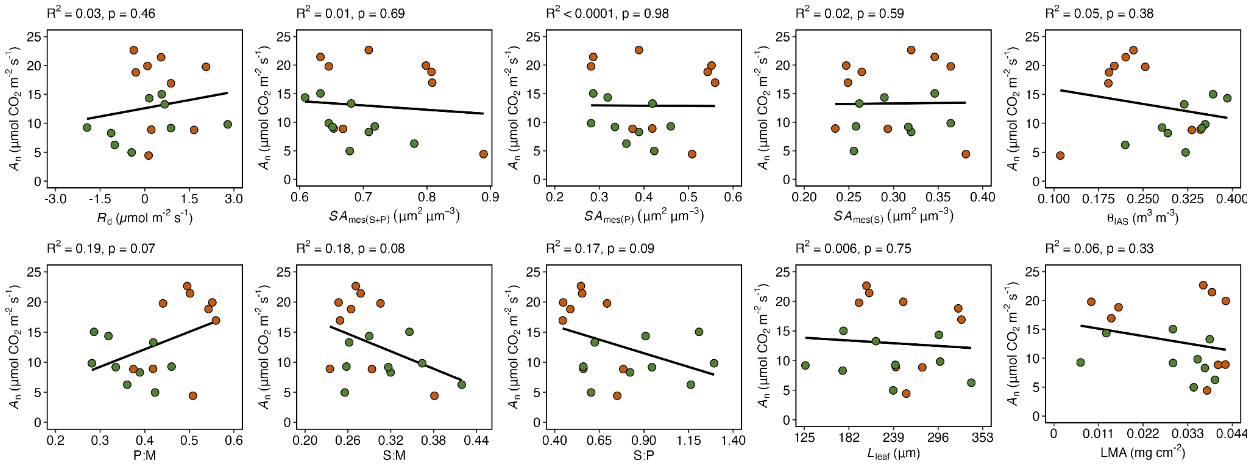

Figure S8. Net assimilation rate (A_n) relationship with biochemical and anatomical characteristics of leaves from 2022. Each point represents an individual tree with color indicating leaf habit (deciduous=orange, evergreen=green). A_n was extracted from $A_n - C_i$ curves at C_a of 400 $\mu mol\ mol^{-1}$ (40.5 Pa). Strength of association was evaluated with Pearson's correlation at $\alpha = 0.05$. Summary output from correlation analyses are provided in Table S5. Associations that were statistically significant are displayed in Fig. 5. Abbreviations along x-axes are defined in Table 1.

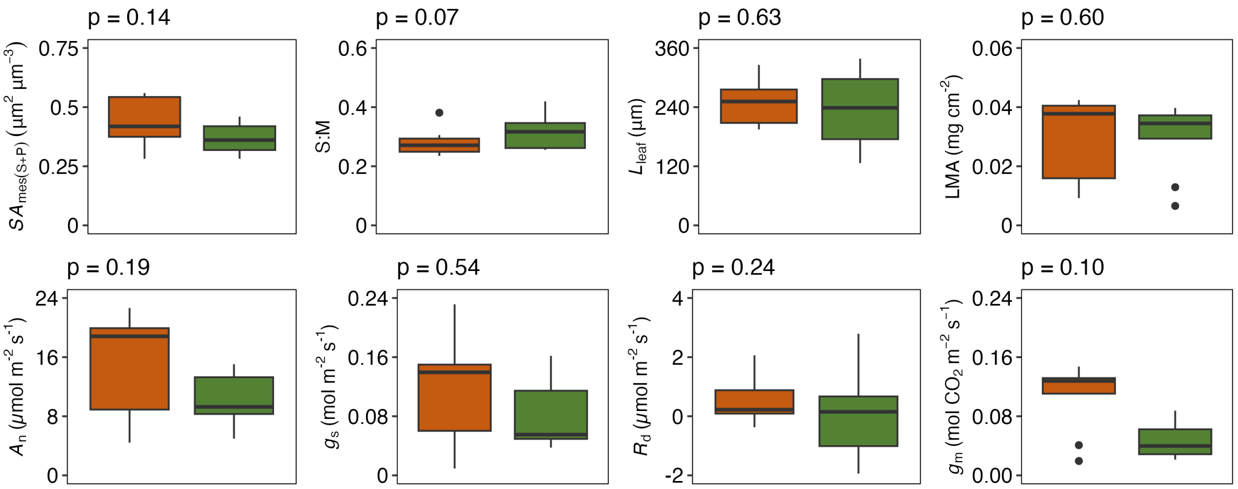

Figure S9. Comparison of biochemical and anatomical characteristics of leaves between leaf habits. Color indicates leaf habit (deciduous=orange, evergreen=green). P-values displayed are from phylogenetic ANOVA testing based on 1,000 residual randomization permutations. Summary output from phylogenetic ANOVA testing is provided in Table S4. Biochemical and anatomical characteristics of leaves that were significantly different between leaf habits are displayed in Figure 4. Abbreviations along x-axes are defined in Table 1. In each boxplot, the box represents the interquartile range spanning from the 25th to 75th percentiles, the horizontal line within the box represents the median, whiskers extend to the minimum and maximum values, and points are outliers. The analysis includes a total of nine measurements (n = 9) for each evergreen and deciduous species group.

Table S1. Collection information for the individual trees of 6 oak species sampled in June 2022 and June 2024 in the Peter J. Shields Oak Grove at the University of California Davis Arboretum and Public Garden (Davis, CA, USA). Tree tag is the tag number on each tree. Scientific name is the genus and species of each tree. Accession number and collection information were provided by the arboretum. Notes indicate findings noted during sample collection and indicate if an individual tree was sampled multiple times to ensure identical replication (n=3 trees) per species.

tree tag	accession number	scientific name	collection information (e.g. year, origin, locality)	notes
26	A65.0838	*Q. rugosa* Née	1963; Huntington Botanical Gardens, San Marino, CA, USA; 3 km before El Oro on road from Atlacomulco, Mexico	
65	A64.1325	*Q. suber* L.	1964; Barcelona, Spain	Sampled twice: leaf samples 65A & 65B taken from same tree
80	A64.1280	*Q. suber* L.	1964; Quad area University of California Davis, Davis, CA, USA	
81	A64.1304	*Q. cerris* L	1964; Royal Botanic Gardens, Kew, Richmond, United Kingdom	Sampled twice: leaf samples 81A & 81B taken from same tree
82	A64.1304	*Q. cerris* L	1964; Royal Botanic Gardens, Kew, Richmond, United Kingdom	
236	A68.0350	*Q. obtusata* Humb. & Bonpl.	1966; 60 miles from Jalapa, Veracruz, Mexico	
238	A68.0350	*Q. obtusata* Humb. & Bonpl.	1966; 60 miles from Jalapa, Veracruz, Mexico	
241	A68.0350	*Q. obtusata* Humb. & Bonpl.	1966; 60 miles from Jalapa, Veracruz, Mexico	
242	A68.0361	*Q. crassipes* Humb. & Bonpl.	1966; 2 mi. southeast of El Seco junction road to	

			Orizaba, Puebla, Mexico	
243	A68.0361	*Q. crassipes* Humb. & Bonpl.	1966; 2 mi. southeast of El Seco junction road to Orizaba, Puebla, Mexico	
253	A68.0361	*Q. crassipes* Humb. & Bonpl.	1966; 2 mi. southeast of El Seco junction road to Orizaba, Puebla, Mexico	
258	A68.0360	*Q. rugosa* Née	1966; 20 miles from Oaxaca on the road to Boone Halberg's finca near Ixtlan, Mexico	
261	A68.0355	*Q. crassifolia* Humb. & Bonpl.	1966; 80 km marker on RN-l between Petzcia and Lago Atitlan, Guatemala	Sampled three times: leaf samples 261A, 261B, & 261C taken from same tree
accession	M10.0002	*Q. rugosa* Née	2010; Near Carretera Cuahimoloyas on ridge by ranch, 2300 m, Oaxaca, Mexico	

Table S2. Precision, accuracy, recall, and F1 scores as a function of annotated leaves for the "big model" with combined replication per species (n = 18 masks; n=12 masks for training and n=6 masks for testing) were used to predict tissue classes in X-ray µCT images.

Species	Tissue class	Precision	Recall	Accuracy	F1
Q. obtusata	Adaxial epidermis	0.88	0.82	0.99	0.84
	Abaxial epidermis	0.83	0.87	0.99	0.85
	Spongy mesophyll	0.82	0.83	0.96	0.81
	Palisade mesophyll	0.83	0.81	0.94	0.81
	Intercellular airspace	0.77	0.78	0.94	0.76
	Bundle sheath	0.75	0.73	0.94	0.73
	Vein	0.86	0.77	0.98	0.81
Q. cerris	Adaxial epidermis	0.85	0.87	0.99	0.86
	Abaxial epidermis	0.85	0.87	0.99	0.86
	Spongy mesophyll	0.83	0.80	0.95	0.81
	Palisade mesophyll	0.87	0.86	0.95	0.86
	Intercellular airspace	0.76	0.78	0.95	0.77
	Bundle sheath	0.71	0.81	0.97	0.76
	Vein	0.83	0.77	0.98	0.80
Q. crassifolia	Adaxial epidermis	0.83	0.81	0.99	0.82
	Abaxial epidermis	0.87	0.80	0.99	0.82
	Spongy mesophyll	0.80	0.83	0.94	0.81
	Palisade mesophyll	0.83	0.86	0.91	0.84

	Intercellular airspace	0.80	0.79	0.93	0.77
	Bundle sheath	0.79	0.76	0.92	0.78
	Vein	0.82	0.82	0.97	0.82
Q. suber	Adaxial epidermis	0.81	0.86	0.98	0.83
	Abaxial epidermis	0.79	0.79	0.98	0.78
	Spongy mesophyll	0.83	0.75	0.96	0.82
	Palisade mesophyll	0.79	0.82	0.94	0.80
	Intercellular airspace	0.79	0.79	0.95	0.79
	Bundle sheath	0.72	0.73	0.95	0.71
	Vein	0.75	0.78	0.97	0.75
Q. crassipes	Adaxial epidermis	0.84	0.89	0.99	0.86
	Abaxial epidermis	0.83	0.83	0.99	0.83
	Spongy mesophyll	0.84	0.84	0.95	0.84
	Palisade mesophyll	0.79	0.81	0.94	0.81
	Intercellular airspace	0.81	0.82	0.95	0.81
	Bundle sheath	0.71	0.73	0.94	0.72
	Vein	0.78	0.77	0.96	0.76
Q. rugosa	Adaxial epidermis	0.78	0.90	0.99	0.83
	Abaxial epidermis	0.81	0.78	0.99	0.80
	Spongy mesophyll	0.80	0.77	0.96	0.82
	Palisade mesophyll	0.79	0.82	0.95	0.81

	Intercellular airspace	0.74	0.80	0.95	0.77
	Bundle sheath	0.77	0.74	0.93	0.72
	Vein	0.78	0.71	0.96	0.73

Table S3. Biochemical, CO_2 diffusional, anatomical, and morphological data for each tree in (A) 2022, and biochemical and CO_2 diffusional data for each tree in (B) 2024.

(A) 2022

tree	θ_{IAS}	S:M	P:M	S:P	A_{max}	V_{cmax}	J_{max}	R_d	TPU	g_m	A_p	LMA	L_{leaf}	A_n	g_s	$SA_{mes(S+P)}$	$SA_{mes(S)}$	$SA_{mes(P)}$	A_{max} (mass)	A_{max} (thickness)
81A	0.22	0.28	0.50	0.55	18.18	68.24	99.35	0.55	6.65	0.14	19.40	0.04	207.95	21.43	0.14	0.63	0.35	0.29	454.5	0.09
81B	0.23	0.27	0.50	0.55	21.66		82.81	-0.37	7.23	0.15	22.06	0.04	204.39	22.65	0.15	0.71	0.32	0.39	541.5	0.10
82	0.25	0.31	0.44	0.69	23.96	89.28	124.70	2.06	8.42	0.13	23.20	0.01	194.86	19.77	0.13	0.65	0.36	0.28	2396	0.12
261A	0.19	0.26	0.54	0.49	38.96	73.53	167.32	-0.30	11.88	0.13	35.94	0.02	321.65	18.82	0.19	0.81	0.26	0.54	1948	0.12
261B	0.20	0.25	0.55	0.45	22.43	67.52	114.96	0.09	7.37	0.13	22.02	0.04	251.44	19.92	0.14	0.80	0.25	0.55	560.7	0.09
261C	0.19	0.25	0.56	0.44		118.23	133.99	0.88		0.11		0.01	325.73	16.93	0.23	0.81	0.25	0.56		
242	0.37	0.35	0.29	1.21		42.23	44.18	0.57		0.02		0.03	174.99	15.04	0.11	0.63	0.35	0.29		
243	0.29	0.32	0.39	0.82	16.10	18.73	64.37	-1.13	4.71	0.05	15.26	0.04	174.00	8.30	0.05	0.71	0.32	0.39	402.5	0.09
253	0.35	0.36	0.28	1.29	14.76	76.21	96.06	2.79	5.63	0.03	14.10	0.04	298.52	9.82	0.04	0.65	0.36	0.28	369	0.05
238_240	0.35	0.23	0.42	0.56	13.88	23.42	59.82	0.22	4.82	0.04	14.24	0.04	241.93	8.90	0.01	0.65	0.23	0.42	347	0.06
236	0.11	0.38	0.51	0.75		58.72	53.17	0.13		0.02		0.04	255.24	4.43	0.04	0.89	0.38	0.51		
241	0.33	0.29	0.37	0.78		59.33	61.15	1.66		0.11		0.04	275.76	8.85	0.06	0.67	0.29	0.37		
10.0002	0.39	0.29	0.32	0.91	12.60	28.62	58.62	0.15	4.25	0.07	12.60	0.01	296.99	14.33	0.16	0.61	0.29	0.32	1260	0.04
26	0.32	0.26	0.42	0.60	6.91	15.95	26.88	-0.44	2.15	0.04	6.89	0.03	238.59	4.97	0.04	0.68	0.26	0.42	230.3	0.03
258	0.22	0.42	0.36	1.16	10.56	16.29	39.34	-1.01	3.31	0.06	10.94	0.04	338.38	6.27	0.06	0.78	0.42	0.36	264	0.03
65A	0.32	0.26	0.42	0.62	11.73	69.14	70.79	0.67	4.34	0.02	12.35	0.04	216.48	13.28	0.05	0.68	0.26	0.42	293.2	0.06
65B	0.28	0.26	0.46	0.56	13.95	27.75	48.98	-1.94	3.98	0.03	13.88	0.01	241.39	9.27	0.10	0.72	0.26	0.46	1395	0.06
80	0.35	0.32	0.33	0.95		38.62	51.59	0.88		0.09		0.03	126.38	9.17	0.13	0.65	0.32	0.33		

(B) 2024

tree	V_{cmax}	J_{max}	R_d	TPU	A_p	A_n	g_s
81A	78.31	96.83	1.77	6.75	18.48	19.9	013
81B	70	100	0.34	5.79	17.03	13.5	0.12
82	94.31	219.34	1.97	14.57	41.74	7.85	0.05
261A	88.65	193.65	1.86	13.39	38.31	24.21	0.21
261B	93.1	227.65	1.88	15.36	44.2	22.23	0.18
261C	78.31	96.83	1.77	6.75	18.48	25.41	0.24
242	31.38	50.3	-0.061	3.2	9.661	6.36	0.03
243	49.15	66.97	2.52	3.81	8.91	5.28	0.02
253	78.13	104.08	3.27	7.06	17.91	8.12	0.04
240	49.11	60.24	1.46	4.36	11.62	7.57	0.03
236	56.08	108.65	1.12	7.31	20.81	7.57	0.04
241	48.61	55.5	0.38	4.63	13.51	7.41	0.05
10.0002	55.86	99.36	1.59	5.07	13.62	10.84	0.06
26	23.74	37.3	2.83	2.72	5.33	2.36	0.05
258	46.27	92.14	2.59			7.19	0.03
65A	47.57	71.67	2.1	4.37	11.01	8.21	0.07
65B	44.38	49.21	0.72	3.31	9.21	6.68	0.04
80	43.06	77.31	1.35	6.05	16.8	9.67	0.10

Table S4. Summary output from phylogenetic ANOVAs of biochemical, CO_2 diffusional, anatomical, and morphological characteristics measured in 2022 between leaf habits (deciduous and evergreen). Significance in bold based on 1,000 residual randomization permutations. Data for biochemical and anatomical characteristics that significantly differed between leaf habits are displayed in Fig. 4 and those that did not significantly differ between leaf habits are displayed in Fig. S9.

	trait	F (1,4)	Z	P-value
anatomical	$SA_{mes(S+P)}$	5.62	1.57	**0.051**
	$SA_{mes(P)}$	3.42	1.19	0.144
	$SA_{mes(S)}$	5.62	1.57	**0.051**
	θ_{IAS}	15.70	2.27	**0.015**
	P:M	10.08	2.06	**0.013**
	S:M	4.29	1.45	0.069
	S:P	8.73	1.96	**0.025**
morphological	L_{leaf}	0.23	-0.36	0.63
	LMA	0.30	-0.24	0.602
biochemical	V_{cmax}	36.15	2.55	**0.005**
	J_{max}	7.96	1.8	**0.018**
	A_{max}	8.42	1.89	**0.013**
	TPU	13.20	2.09	**0.013**
	A_p	10.17	1.99	**0.013**
	R_d	1.74	0.73	0.241

	A_n	1.86	0.73	0.187
diffusional	g_s	0.41	-0.07	0.535
	g_m	3.91	1.24	0.104

Table S5. Summary output from correlation analyses between net assimilation rate (A_n) and biochemical, CO_2 diffusional, and anatomical characteristics. Strength of association was evaluated using Pearson's correlation at α = 0.05. Significant associations are displayed in Fig. 5, and those not significant are displayed in Fig. S8.

Correlations with A_n				
Characteristic	t value	F-statistic on 1 and 16 DF	Multiple R-squared	P-value
$SA_{mes(S+P)}$	-0.405	0.1642	0.01016	0.6907
$SA_{mes(P)}$	-0.026	0.0007002	4.376e-05	0.9792
$SA_{mes(S)}$	-0.543	0.2953	0.01812	0.5944
θ_{IAS}	-0.912	0.8325	0.04946	0.3751
P:M	1.95	3.805	0.1921	0.06883
S:M	-1.869	3.493	0.1792	0.08004
S:P	-1.78	3.174	0.1656	0.09378
L_{leaf}	-0.321	0.1033	0.006418	0.752
LMA	-1.008	1.016	0.0597	0.3285
V_{cmax}	3.156	9.962	0.3837	**0.006114**
J_{max}	4.048	16.38	0.5059	**0.0009337**
A_{max}	3.145	9.892	0.4735	**0.00932**
TPU	3.935	15.49	0.5847	**0.00233**
g_m	4.589	21.06	0.57	**0.0003023**
A_p	3.674	13.49	0.5509	**0.003667**
R_d	0.752	0.5648	0.0341	0.4633
g_s	4.47	19.94	0.5548	**0.0003907**

Table S6. Net assimilation rate (A_n) response to intercellular airspace CO_2 concentrations (C_i), estimated assimilation rate at the Rubisco limiting state (A_c), and assimilation rate at the RuBP-regeneration limiting state (A_j) using "Plantecophys" R package at various ambient CO_2 concentrations (C_a) for all six *Quercus* species (3 deciduous and 3 evergreen) collected in 2022 and 2024.

Species	Replication	2022					2024				
		C_a	A_n	C_i	A_c	A_j	C_a	A_n	C_i	A_c	A_j
Q. crassipes	QUCR253	50	-0.69	60.08	0.36	0.53	50	-0.88	75.80	2.01	2.84
		100	-1.82	101.55	3.19	3.94	80	-0.09	78.71	2.28	3.18
		150	3.98	102.09	3.22	3.97	100	0.62	85.69	2.94	3.98
		250	9.15	223.07	7.16	7.16	150	2.26	107.64	4.95	6.13
		400	9.82	165.02	8.47	7.97	200	4.05	123.18	6.33	7.41
		600	11.36	187.40	10.46	.9.04	250	4.63	135.60	7.41	8.32
		800	13.84	847.97	33.47	14.83	400	8.12	179.92	10.92	10.83
		1200	13.71	1024.70	37.43	15.31	600	12.53	177.93	11.08	10.93
		1600	14.76	1369.33	43.74	15.91	800	15.65	356.49	23.49	16.39
							1000	16.67	481.22	30.58	18.28
							1200	16.88	789.59	44.07	20.71
							1600	20.09	899.63	47.88	21.21
							1800	18.06	1167.56	55.60	22.08

	QUCR243	50	-0.55	35.60	-0.86	0.00	50	-1.23	80.82	1.39	1.77
		100	1.21	79.80	1.05	2.16	80	-0.34	92.11	1.98	2.41
		150	3.21	114.69	2.46	4.39	100	-0.08	99.12	2.05	2.48
		250	3.98	211.89	5.99	8.05	150	0.86	108.16	2.34	2.76
		400	8.30	218.66	6.22	8.22	200	1.55	111.50	2.80	3.19
		600	9.70	456.74	12.93	11.71	250	2.31	93.50	2.97	3.34
		800	11.62	678.96	17.56	13.06	400	5.28	141.82	4.46	4.52
		1200	16.91	945.28	21.79	13.94	600	7.27	182.59	6.34	5.72
		1600	15.27	1355.13	26.49	14.66	800	8.04	328.12	12.19	8.18
							1000	8.40	511.90	18.06	9.64
							1200	9.12	755.86	24.05	10.62
							1600	8.89	972.06	28.18	11.12
							1800	9.28	1097.81	30.21	11.33
	QUCR242	50	-0.80	52.82	-0.13	0.00	50	0.02	47.53	-0.32	0.00
		100	0.96	84.72	1.78	1.97	80	0.79	47.33	-0.31	0.00
		150	3.25	108.55	3.15	3.16	100	0.41	84.50	1.22	1.81
		250	6.54	182.20	7.06	5.61	150	1.76	100.09	1.84	2.57
		400	8.32	315.52	13.10	7.90	200	2.65	126.85	2.87	3.63
		600	7.09	508.44	20.05	9.46	250	4.01	156.67	3.97	4.56
		800	8.53	562.51	21.71	9.74	400	6.36	194.88	5.30	5.49
		1200	10.24	1088.01	33.55	11.12	600	7.34	214.08	5.94	5.88
		1600	12.78	1274.38	36.49	11.36	800	8.81	295.41	8.46	7.10
							1000	8.65	498.12	13.60	8.72
							1200	9.23	694.23	17.47	9.52
							1600	10.80	988.46	21.91	10.17
							1800	10.01	1178.49	24.17	10.44

Q. obtusata	QUOB241	50	-2.34	54.94	-0.00	0.00	50	-0.21	56.66	0.12	0.16
		100	1.26	76.72	1.90	2.07	80	0.56	62.57	0.56	0.71
		150	3.84	124.02	5.82	5.25	100	0.73	80.05	1.85	2.15.
		250	6.38	180.37	10.11	7.68	150	2.29	94.23	2.86	3.14
		400	8.85	240.90	14.32	9.43	200	3.69	108.04	3.83	3.97
		600	8.07	357.22	21.41	11.51	250	4.50	124.11	4.92	4.82
		800	10.20	481.26	27.82	12.83	400	7.41	239.11	11.98	8.61
		1200	15.64	912.04	43.93	14.92	600	9.46	307.40	15.63	9.88
		1600	13.05	1267.73	52.80	15.67	800	11.28	472.90	23.14	11.71
							1000	12.37	650.68	29.62	12.79
							1200	13.27	805.70	34.29	13.39
							1600	14.30	933.97	37.61	13.75
							1800	12.76	1138.90	42.12	14.18
	QUOB240	50	-0.14	50.08	-0.16	0.00	50	-0.62	64.72	0.61	0.85
		100	0.47	80.45	0.84	2.04	80	0.07	75.51	1.29	1.69
		150	2.63	118.58	1.05	4.24	100	0.68	86.65	1.97	2.46
		250	4.42	242.24	5.49	8.11	150	2.04	114.75	3.63	4.07
		400	5.97	287.27	6.59	8.92	200	3.91	122.54	4.08	4.45
		600	10.20	437.44	9.78	10.66	250	4.72	142.50	5.20	5.31
		800	9.06	698.44	14.07	12.15	400	7.57	183.45	7.39	6.71
		1200	14.72	970.34	17.37	12.93	600	8.85	273.38	11.74	8.73
		1600	13.96	1322.62	20.55	13.51	800	9.68	442.92	18.55	10.75
							1000	10.17	672.70	25.70	12.10
							1200	11.67	870.36	30.51	12.75
							1600	12.34	965.61	32.50	12.98
							1800	12.30	1165.22	36.12	13.34

	QUOB238	50	-0.60	62.80	-0.43	0.00	50	-0.36	61.49	0.30	0.89
		100	1.46	82.04	2.20	2.22	80	0.37	64.95	0.46	1.33
		150	2.60	119.43	5.33	4.63	100	0.61	78.44	1.07	2.92
		250	4.72	210.40	12.12	8.08	150	2.03	82.34	1.25	3.34
		400	8.90	289.27	17.11	9.74	200	2.89	110.04	2.25	5.88
		600	8.76	523.07	26.61	11.85	250	4.40	119.21	2.84	6.58
		800	10.00	438.60	36.53	13.26	400	7.57	279.67	8.79	13.55
		1200	16.40	1033.44	45.12	14.12	600	10.03	321.84	10.13	14.53
		1600	13.88	1327.75	53.47	14.12	800	13.35	490.46	14.77	17.05
						14.75	1000	16.24	530.23	15.73	17.45
							1200	19.19	623.34	17.79	18.22
							1600	18.83	868.08	22.27	19.54
							1800	20.83	1056.00	25.02	20.18
Q. suber	QUSU65A	50	-0.76	54.17	-0.06	0.00	50	-1.03	93.57	1.15	1.85
		100	1.63	77.516	1.74	2.88	80	-0.44	106.53	1.36	2.14
		150	3.72	128.52	5.45	4.83	100	0.28	80.02	2.07	3.08
		250	9.80	149.85	6.91	5.72	150	1.50	76.15	2.74	3.87
		400	13.28	182.75	9.07	6.83	200	2.37	108.84	2.86	4.00
		600	12.65	473.62	24.20	11.24	250	3.54	156.95	5.20	6.21
		800	13.31	593.96	28.93	11.99	400	8.21	234.92	8.44	8.38
		1200	12.82	952.73	39.75	13.23	600	10.68	230.03	8.65	8.49
		1600	10.63	1212.19	45.47	13.71	800	11.04	341.68	12.76	10.33
							1000	11.16	503.29	17.93	11.90
							1200	11.09	653.80	21.89	12.76
							1600	11.46	704.30	23.07	12.98
							1800	10.31	981.70	28.53	13.81

	QUSU65B	50	-0.36	43.42	-0.47	0.00	50	-0.44	57.22	0.12	0.16
		100	1.78	65.14	0.19	0.39	80	-0.14	78.95	1.31	1.52
		150	5.03	59.69	0.40	0.83	100	1.14	83.61	1.56	1.78
		250	11.07	162.02	3.98	5.65	150	2.68	112.05	3.03	3.09
		400	9.26	295.69	8.10	8.53	200	4.70	115.13	3.19	3.22
		600	12.57	346.05	9.45	9.17	250	6.22	178.00	6.20	5.15
		800	13.25	679.85	16.47	11.37	400	6.68	257.96	8.08	6.03
		1200	14.53	830.66	18.84	11.85	600	6.95	272.71	9.63	6.65
		1600	13.95	1214.13	23.48	12.58	800	7.75	220.51	10.22	6.85
							1000	9.00	447.24	16.33	8.48
							1200	8.67	699.84	23.07	9.60
							1600	9.64	966.90	28.39	10.20
							1800	9.59	1070.83	30.11	10.37
	QUSU80	50	-0.51	58.93	0.24	0.35	50	-0.39	59.24	0.27	0.55
		100	0.71	89.54	2.09	2.66	80	-0.24	81.96	1.69	3.07
		150	2.42	110.45	3.30	3.86	100	0.76	85.32	1.90	3.40
		250	5.61	186.50	7.36	6.81	150	2.08	117.05	3.78	5.98
		400	9.17	312.35	13.11	9.36	200	3.96	148.56	5.56	7.92
		600	5.39	485.51	19.53	11.11	250	5.93	174.43	6.95	9.19
		800	10.92	652.66	21.56	11.52	400	9.67	250.16	10.73	11.86
		1200	13.58	549.48	24.52	12.04	600	12.67	327.89	14.18	13.64
		1600	13.06	1200.65	35.77	13.43	800	13.39	405.18	16.58	14.63
							1000	14.31	387.34	17.26	14.88
							1200	15.76	675.17	25.90	17.29
							1600	16.77	781.05	28.61	17.84
							1800	16.82	1062.78	34.52	18.81

Q. cerris	QUCE81A	50	-0.57	64.72	0.92	1.38	50	-1.1	54.83	-0.02	0.00
		100	2.31	73.44	1.74	2.50	80	0.7	72.03	2.13	3.62
		150	5.00	109.91	5.05	6.21	100	2.0	82.57	2.42	5.53
		250	10.94	180.44	10.70	10.73	150	4.7	110.76	6.75	9.76
		400	14.71	237.15	13.06	13.04	200	8.4	132.49	9.22	12.35
		600	16.22	316.86	15.27	15.25	250	11.0	162.54	12.49	15.25
		800	16.89	678.62	19.14	19.11	400	19.9	239.41	20.18	20.47
		1200	20.15	997.90	19.68	19.66	600	26.9	358.44	30.49	25.04
		1600	21.21	604.28	21.18	21.15	800	29.8	456.84	37.81	27.41
							1000	28.1	473.22	38.94	27.72
							1200	28.2	727.24	53.86	31.04
							1600	30.0	833.92	58.95	31.90
							1800	28.6	1097.82	69.42	33.38
	QUE81B	50	-0.87	64.93	0.90	1.53	50	-1.1	63.67	0.92	1.27
		100	2.56	65.54	0.95	1.62	80	0.6	70.51	1.64	2.19
		150	5.68	99.89	3.96	5.77	100	1.3	84.46	3.08	2.86
		250	11.15	191.27	11.17	12.20	150	4.4	108.51	5.49	6.24
		400	16.89	235.12	14.28	14.08	200	7.4	138.75	8.38	8.58
		600	21.69	487.01	28.79	19.52	250	9.7	169.40	11.17	10.43
		800	22.19	608.31	34.25	20.77	400	13.5	247.80	17.73	13.73
		1200	20.38	866.90	43.63	22.41	600	14.7	347.87	25.07	16.30
		1600	22.92	1415.81	57.13	24.05	800	17.6	395.80	28.23	17.17
							1000	17.4	466.72	32.56	18.29
							1200	21.2	599.57	39.68	19.56
							1600	16.6	712.43	44.91	20.37
							1800	17.7	872.63	51.31	21.20

	QUCE82	50	-0.52	64.58	1.09	1.61	50	-0.69	66.43	0.92	1.27
		100	1.62	81.14	2.93	4.03	80	0.70	76.70	1.64	2.19
		150	4.61	115.16	6.55	7.87	100	1.06	94.80	3.08	3.86
		250	11.58	161.60	11.19	11.56	150	2.96	123.12	5.49	6.24
		400	19.76	231.20	17.52	15.22	200	3.95	143.99	8.38	8.58
		600	18.07	396.94	30.22	19.888	250	5.18	164.76	11.17	10.43
		800	21.75	639.82	44.44	22.98	400	7.85	229.58	17.73	13.73
		1200	24.76	902.05	55.91	24.66	600	8.57	355.78	25.07	16.30
		1600	23.15	1353.96	69.91	26.14	800	12.26	433.81	28.23	17.17
							1000	13.85	689.15	32.56	18.19
							1200	15.49	750.48	39.68	19.56
							1600	18.46	903.74	44.91	20.37
							1800	17.03	1180.27	51.31	21.20
Q. rugosa	QURU258	50	-1.00	175.72	0.13	0.34	50	-1.16	91.03	2.45	1.70
		100	3.32	72.53	0.33	0.83	80	-0.31	92.44	2.54	4.85
		150	2.28	67.68	0.46	1.11	100	0.04	94.68	2.69	5.09
		250	3.31	201.12	3.48	5.46	150	1.18	104.58	3.33	6.06
		400	6.26	269.53	4.85	6.56	200	2.15	138.90	4.36	7.48
		600	8.99	443.29	7.80	8.15	250	3.74	120.82	5.48	8.85
		800	9.83	613.84	10.10	8.97	400	7.19	245.33	11.44	14.03
		1200	11.69	884.72	12.4	9.69	600	11.73	280.71	13.22	15.16
		1600	10.55	1373.39	16.49	10.32	800	15.62	482.28	21.83	18.94
							1000	18.13	686.11	28.56	20.81
							1200	20.00	803.23	31.76	21.50
							1600	18.37	918.16	34.55	22.03
							1800	20.79	1180.54	39.86	22.89

	QURU10.0002	50	0.95	38.40	-1.39	0.00	50	-0.93	62.56	0.49	0.85
		100	2.84	83.40	2.29	2.47	80	-0.44	125.45	3.06	4.47
		150	6.79	97.19	3.36	3.42	100	-0.14	103.89	3.23	4.66
		250	7.89	171.16	8.72	6.94	150	1.33	106.65	4.34	5.85
		400	12.91	298.40	16.61	10.02	200	3.63	126.36	4.39	5.91
		600	14.33	505.59	26.76	12.30	250	5.54	158.84	6.23	7.56
		800	13.31	548.90	28.56	12.60	400	10.84	238.10	10.33	10.29
		1200	13.62	654.17	32.56	13.18	600	14.84	272.84	11.97	11.13
		1600	11.57	1025.0	43.49	14.36	800	13.42	358.28	15.69	12.67
							1000	9.88	451.31	19.27	13.81
							1200	10.56	579.71	23.56	14.88
							1600	15.68	800.80	29.58	16.02
							1800	12.46	1097.18	35.74	16.89
	QURU26	50	0.23	43.00	-0.28	0.00	50	-1.20	123.95	2.00	2.83
		100	1.26	74.77	0.45	0.85	80	-0.47	174.14	2.07	2.90
		150	1.32	114.27	1.30	2.11	100	-0.31	121.42	2.22	3.06
		250	3.15	196.51	2.91	3.69	150	0.19	129.36	2.93	3.72
		400	4.97	248.83	3.48	4.32	200	0.44	155.09	2.99	3.77
		600	4.55	263.71	4.09	.4.46	250	0.83	157.12	3.44	4.13
		800	7.89	468.03	7.03	5.71	400	2.36	180.58	3.61	4.26
		1200	7.89	630.56	8.88	6.21	600	3.74	256.91	5.47	5.43
		1600	4.95	933.03	11.52	6.73	800	4.20	396.94	8.40	6.65
							1000	5.99	495.82	10.16	7.18
							1200	5.25	646.72	12.45	7.71
							1600	6.01	790.38	14.30	8.06
							1800	4.15	890.61	15.43	8.24

Q. crassifolia	QUCR261A	50	1.75	47.86	-0.79	0.00	50	-1.38	54.43	-0.08	0.00
		100	2.32	57.86	0.31	0.74	80	0.76	74.84	2.75	5.64
		150	3.64	121.24	6.41	11.79	100	2.18	86.75	4.36	8.45
		250	11.30	115.90	6.94	12.53	150	6.32	117.59	8.38	14.39
		400	18.81	286.20	21.35	25.53	200	9.70	155.30	13.03	19.79
		600	30.47	381.31	28.19	29.05	250	13.95	171.42	14.93	21.65
		800	34.85	471.84	33.92	31.34	400	24.21	271.01	25.68	29.67
		1200	29.99	517.20	26.56	32.24	600	34.37	424.59	39.50	36.18
		1600	35.95	839.54	51.71	36.14	800	39.50	546.40	48.61	39.18
							1000	41.12	711.10	58.98	41.81
							1200	41.18	848.05	66.27	43.32
							1600	42.74	1105.52	77.50	45.23
							1800	41.31	1111.23	77.72	45.26
	QUCR261B	50	0.85	32.53	-2.33	0.00	50	-1.55	54.65	0.04	0.00
		100	1.63	83.16	2.80	4.01	80	0.73	73.81	2.47	5.02
		150	4.42	108.94	5.24	6.77	100	2.18	85.84	4.01	7.73
		250	10.35	186.82	12.04	12.21	150	5.97	115.75	7.70	13.21
		400	17.99	240.15	16.24	14.53	200	8.95	146.99	11.37	17.57
		600	19.92	329.41	22.56	17.17	250	12.49	179.82	15.03	21.14
		800	21.20	644.73	39.69	21.48	400	22.23	261.40	23.35	27.28
		1200	23.65	986.49	.52.39	23.35	600	31.30	390.36	34.65	32.90
		1600	21.21	1051.54	54.35	23.58	800	34.76	534.86	45.18	36.53
							1000	36.24	595.83	49.08	37.61
							1200	36.09	606.41	49.73	37.78
							1600	38.87	780.16	59.34	39.99
							1800	37.76	900.65	65.02	41.08

	QUCR261C	50	-0.26	49.50	-0.85	0.00	50	-1.50	54.84	-0.02	0.00
		100	2.12	79.04	3.65	4.67	80	0.84	73.91	2.60	5.63
		150	6.07	107.40	7.78	8.85	100	2.34	87.67	4.45	9.05
		250	16.10	148.32	13.40	13.26	150	6.73	115.91	8.10	14.77
		400	16.92	308.47	32.25	22.23	200	9.97	152.84	12.62	20.39
		600	23.43	425.93	43.55	25.40	250	14.24	188.07	16.70	24.45
		800	26.14	647.16	60.68	28.71	400	25.41	282.19	26.56	31.73
		1200	33.44	957.89	78.51	31.03	600	36.31	447.77	40.98	38.57
		1600	33.79	1351.43	94.59	32.56	800	41.46	598.39	51.66	42.01
							1000	42.93	725.53	59.29	43.95
							1200	43.47	839.29	65.26	45.25
							1600	45.27	969.69	71.31	46.40
							1800	43.12	1105.90	76.85	47.33
